# The venom and telopodal defence systems of the centipede *Lithobius forficatus* are functionally convergent serial homologues

**DOI:** 10.1186/s12915-024-01925-x

**Published:** 2024-06-13

**Authors:** Vanessa Schendel, Carsten H. G. Müller, Matthes Kenning, Michael Maxwell, Ronald A. Jenner, Eivind A. B. Undheim, Andy Sombke

**Affiliations:** 1 Centre for Advanced Imaging, The University of Queensland, St. Lucia, QLD 4072 Australia; 2 Institute for Molecular Bioscience, The University of Queensland, St. Lucia, QLD 4072 Australia; 3 Zoological Institute and Museum, University of Greifswald, Loitzer Strasse 26, Greifswald, 17489 Germany; 4 Natural History Museum, Cromwell Road, London, SW7 5BD UK; 5 Centre for Ecological and Evolutionary Synthesis, Department of Biosciences, University of Oslo, Oslo, 0316 Norway; 6 Centre for Anatomy and Cell Biology, Cell and Developmental Biology, Medical University of Vienna, Schwarzspanierstrasse 17, Vienna, 1090 Austria; 7 Department of Evolutionary Biology, Integrative Zoology, University of Vienna, Djerassiplatz 1, 1030 Austria

**Keywords:** Epidermal exocrine glands, Evolution, Chilopoda, Myriapoda, Arthropoda, Venom, Novelty, Innovation, Telopodal glandular organs

## Abstract

**Background:**

Evolution of novelty is a central theme in evolutionary biology, yet studying the origins of traits with an apparently discontinuous origin remains a major challenge. Venom systems are a well-suited model for the study of this phenomenon because they capture several aspects of novelty across multiple levels of biological complexity. However, while there is some knowledge on the evolution of individual toxins, not much is known about the evolution of venom systems as a whole. One way of shedding light on the evolution of new traits is to investigate less specialised serial homologues, i.e. repeated traits in an organism that share a developmental origin. This approach can be particularly informative in animals with repetitive body segments, such as centipedes.

**Results:**

Here, we investigate morphological and biochemical aspects of the defensive telopodal glandular organs borne on the posterior legs of venomous stone centipedes (Lithobiomorpha), using a multimethod approach, including behavioural observations, comparative morphology, proteomics, comparative transcriptomics and molecular phylogenetics. We show that the anterior venom system and posterior telopodal defence system are functionally convergent serial homologues, where one (telopodal defence) represents a model for the putative early evolutionary state of the other (venom). Venom glands and telopodal glandular organs appear to have evolved from the same type of epidermal gland (four-cell recto-canal type) and while the telopodal defensive secretion shares a great degree of compositional overlap with centipede venoms in general, these similarities arose predominantly through convergent recruitment of distantly related toxin-like components. Both systems are composed of elements predisposed to functional innovation across levels of biological complexity that range from proteins to glands, demonstrating clear parallels between molecular and morphological traits in the properties that facilitate the evolution of novelty.

**Conclusions:**

The evolution of the lithobiomorph telopodal defence system provides indirect empirical support for the plausibility of the hypothesised evolutionary origin of the centipede venom system, which occurred through functional innovation and gradual specialisation of existing epidermal glands. Our results thus exemplify how continuous transformation and functional innovation can drive the apparent discontinuous emergence of novelties on higher levels of biological complexity.

**Supplementary Information:**

The online version contains supplementary material available at 10.1186/s12915-024-01925-x.

## Background

Evolution of novelty is a central theme in evolutionary biology that seeks to understand the origins of the traits that form the foundation of adaptation [1–7]. Elucidating how novelties evolve can be challenging, especially for traits that lack known homologues that can represent putative intermediate evolutionary steps. This is also the case for venom, which has evolved on more than 100 occasions throughout the animal kingdom [8]. Venom systems are complex traits comprising structures for injecting venom, tissues for producing venom, as well as unique mixtures of bioactive molecules, called toxins. These toxins consist mainly of proteins and peptides that have evolved from non-toxin ancestors and subsequently undergone extensive functional and structural diversification [4, 8]. Although general trends in the molecular evolution of toxins are relatively well understood [4], the processes by which venom systems emerge as evolutionary novelties remain largely unknown. One approach to trying to overcome this challenge is to leverage the serial homology of traits—homology of repetitive structures in the same organism [9]—that exhibit different degrees of specialisation. According to this approach, light might be shed on the origins of the most specialised serial homologue in a series by interpreting less specialised serial homologues as possible hypothetical evolutionary intermediates. This approach can provide a fruitful guide for further research if both the structure and function of a less specialised serial homologue can be plausibly interpreted as a hypothetical intermediate step in a phylogenetic scenario for the origin of the more specialised serial homologue.

An animal group that is particularly amenable for comparative studies of serially homologous traits are arthropods, whose body plan consists of numerous segments, with some segments being specialised and tagmatised. Arthropods also include numerous venomous taxa, one of which are the centipedes (Chilopoda). Centipedes are among the most ancient terrestrial venomous lineages and comprise five extant orders whose last common ancestor existed well over 400 million years ago [10–12]. One of their distinguishing features that evolved in the last common centipede ancestor is the possession of an anterior pair of specialised venomous appendages called forcipules, which have evolved from the first pair of trunk appendages [13]. Each forcipule bears a venom gland that produces a diverse cocktail of protein and peptide toxins [14]. Interestingly, centipede venom glands are composite glands, consisting of numerous units that are arranged around a chitinous venom duct [15–19]. Dugon et al. [20] studied embryonic stages of centipedes and showed that the centipede venom duct may have evolved from an invagination of the cuticle. It has further been speculated that venom gland units have derived from solitary epidermal glands [15, 18]. However, except for a recapitulation during embryogenesis [20], no intermediate evolutionary stages of this functional and morphological transformation of the appendage are known from adult extant specimens, nor from the fossil record.

While the forcipules are probably the most recognisable of centipede traits, they are not the only evolutionarily innovative centipede appendages. In many centipede species, the last pair of legs, the so-called ultimate legs, are easily noticeable by their unique shape and the way they are positioned and moved in relation to the body. Centipede ultimate legs are also functionally diverse, ranging from claw-like grasping legs used in physical defence, to peculiar stridulating acoustic warning legs, and even to ‘reverse antenna-like’ sensory appendages [21, 22]. One modification of centipede ultimate legs is the possession of aggregates of pores associated with epidermal glands that essentially transform the leg into a predominantly glandular organ [21–23]. These telopodal glands or defence glands, here called telopodal glandular organs, are only known to occur in lithobiomorph centipedes, such as the common European stone centipede *Lithobius forficatus* [22]. They are present on the last four pairs of legs, but are particularly prominent in the ultimate legs [22, 24–28]. In the only anatomical investigation of telopodal gland units, Keil [26] noted that their morphology might be very similar to that of venom gland units. When facing a threat, numerous gland units constituting the telopodal glandular organs secrete a sticky, slowly hardening substance forming short threads that aggregate to thicker filaments that can be several times the length of the centipede [29, 30]. The secretion may also have toxic properties against potential attackers such as ants, which have been observed to become paralysed and eventually die upon contact with the secretion [31]. Thus, telopodal glandular organs are thought to be effective in predator deterrence by immobilising attackers and allowing the centipede to escape [30].

Given the epidermal location and origin of the telopodal gland system as well as, in particular, the hypothesised origin of the centipede venom glands from a former cuticular pore field by invagination (cf. [18]), these two systems may thus be considered as potential serial homologues. This possible serial homology, along with their convergent functional roles as chemical weapons, and the similarity of the telopodal glandular organs to hypothesised early stages in centipede venom gland evolution prompted us to further investigate their evolutionary origins. Here, we use a combination of morphological, behavioural and molecular approaches to investigate the following hypotheses: (1) the telopodal system is used as a defensive weapon. (2) The telopodal defence system and venom system in *L. forficatus* are serial homologues, which (2a) have evolved from appendages bearing the same type of solitary epidermal glands (morphological level) and (2b) produce secretions that have convergently evolved similar functions (biomolecular level). (3) The telopodal glandular organ (aggregated and specialised epidermal glands) is a hypothetical intermediate state in the evolution of the venom gland (internalised gland with venom duct). We further discuss how our findings shed light on the evolution of novelty.

## Results

### The telopodal glandular organs play a role in defence

**Fig. 1:**
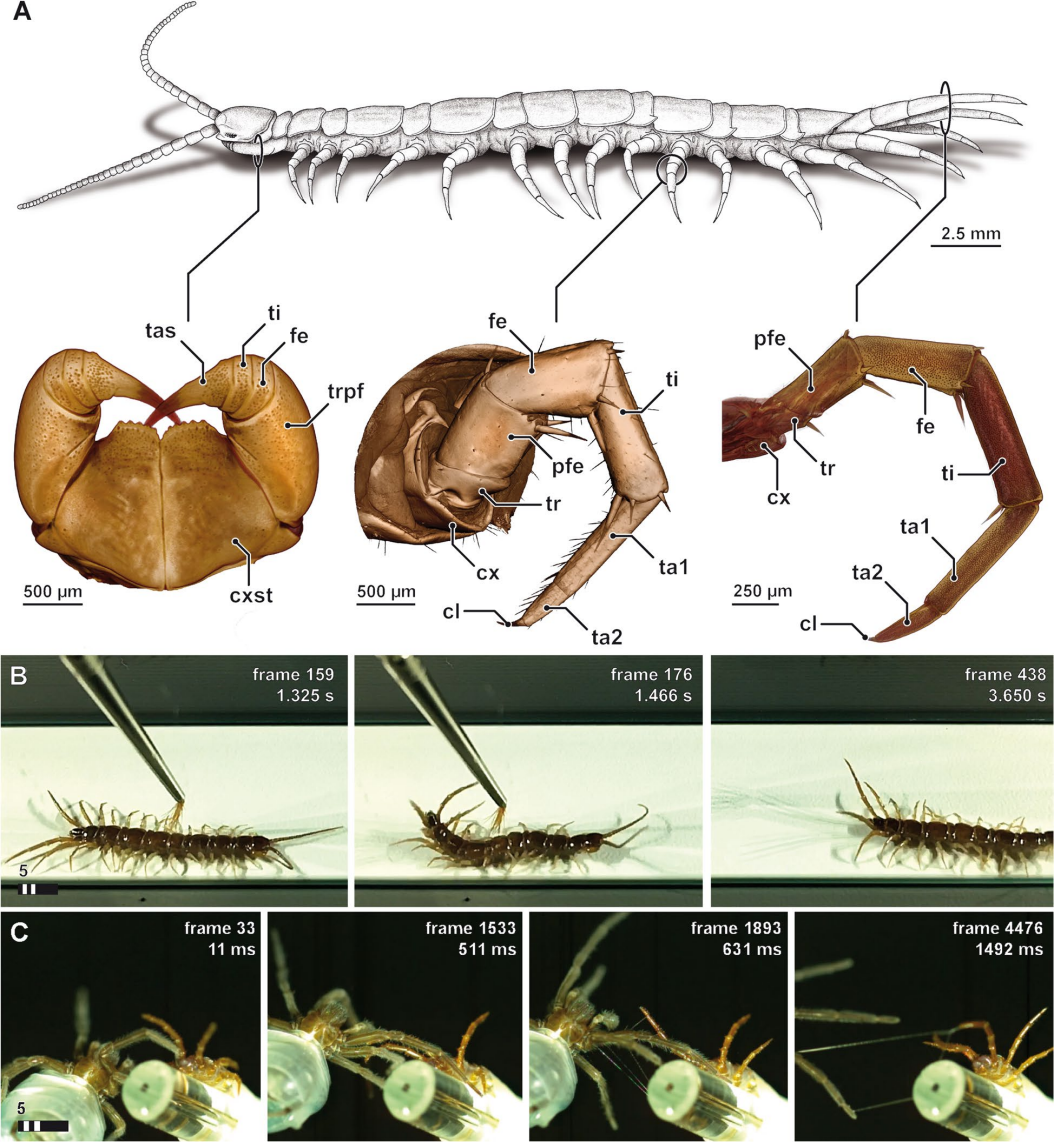
Habitus and defensive display of *Lithobius forficatus*. **A** Habitus of *L. forficatus* and three typical, serially homologous appendages: forcipules (left), locomotory leg 10 (centre) and the ultimate legs (right). 3D volume renderings based on microCT analyses, not to scale. **B** Single frames from high-speed footage (compare Additional file 1) showing the stereotypical defensive display using the tip of a brush. The fast up and down movements of the ultimate legs might also induce or support the distribution of the secretion (compare Additional file 1). Upon contact (left) ultimate and penultimate legs soar up within a tenth of a second in order to present the ventral and medioventral faces of the legs towards the point of irritation (middle). The upright posture of the ultimate legs is maintained for a couple of seconds while the penultimate legs are lowered in order to assist in the escape movement (right). **C** Single frames from high-speed footage (compare Additional file 2) showing the staged encounter of *L. forficatus* (right) and a male lycosid spider (left). Already shortly before contact, *L. forficatus* raises leg pairs 13–15 (leg 12 and more anterior legs were strapped down; left and middle left). Upon contact, beads-on-a-string-like threads become visible, emanating from and connecting ultimate legs and the spider (middle right and right). cl claw, cx coxa, cxst coxosternite, fe femur, pfe prefemur, ta1 tarsus 1, ta2 tarsus 2, tas tarsungulum, ti tibia, tr trochanter, trpf trochanteroprefemur

To confirm the defensive role of the telopodal glandular organs of the ultimate leg pair and its secretion, we conducted several behavioural observations under staged and semi-staged conditions with and without the use of high-speed camera. In any encounter experienced as threatening, *L. forficatus* (Fig. 1A) displays a clear defensive behaviour: leg pairs 12–15 quickly swing upwards, and the ultimate legs (leg pair 15) take an almost perpendicular posture for up to 20 s, followed either by an attempt to sting using the forcipules, or escape with the ultimate legs kept elevated (Fig. 1B; video in Additional file 1). During this leg display behaviour, the telopodal glandular organs release small droplets of a sticky secretion that upon physical contact result in the formation of numerous beads-on-a-string-like threads that can reach a length of at least 3 cm between aggressor and the centipede’s legs (Fig. 1C; video in Additional file 2). If the centipede is still unable to reach the source of irritation, trunk and legs rapidly shake sideways, presumably promoting the dispersion of the secretion. Although the secretion has been described previously as being sprayed across some distance (cf. [31]), our behavioural observations suggest that this is probably not the case. Instead, the secretion appears to be hurled rather than sprayed towards the aggressor (cf. [29]), although it is most likely that it is the aimed grasping movements of the last couple of leg pairs that is the primary mode by which the secretion makes contact with the target. These movements of the legs and trunk result in the formation of numerous sticky threads that detach from the centipede’s legs upon drying and leave the aggressor immobilised.

### Morphology suggests that telopodal glandular organs and venom glands are serial homologues

**Fig. 2:**
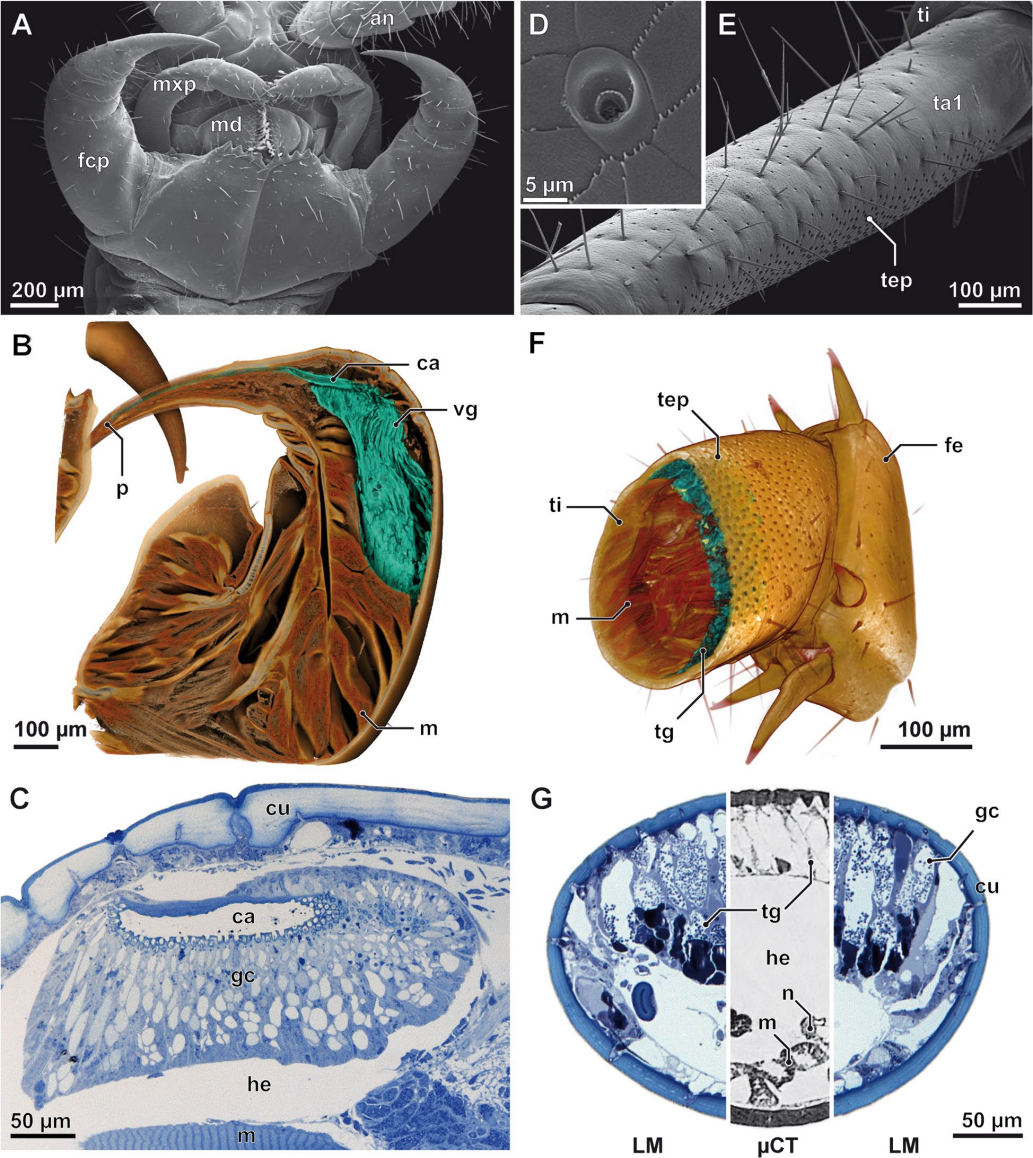
The telopodal glandular organs share a common organisation with venom glands. Morphology and glandular histology of the forcipule (**A**–**C**) and the ultimate leg (**D**–**G**). SEM micrographs of the ventral head with (**A**) forcipules and (**E**) the ventral aspect of the right ultimate leg tarsus 1 (ta1). The lateral face of the tarsus is covered by trichoid sensilla of various lengths, while the medial face is covered by pores of telopodal gland units (tep). **D** Close-up of a pore of a telopodal gland unit showing its non-symmetrical morphology: the shallow part of the pore always faces towards the distal tip of the leg. **B** 3D volume rendering of the left forcipule with labelled venom gland (vg) and associated calyx (ca), and **F** of the proximal femur (fe) and distal tibia (ti) of the ultimate leg with telopodal gland units (tg; medial face to front). Glandular tissue in both appendages highlighted in turquoise. Note that critical point drying affected the structural integrity of the telopodal glandular tissue (compare also shrinkage in **G**). **C** Longitudinal section of the forcipule and calyx (ca) showing the densely packed venom gland units (gc) individually connected to the cuticular calyx. **G** Cross section of the ultimate leg tibia, medial face to top. Blue-stained aspects based on histological sections, grey-stained aspect based on microCT analysis. After chemical fixation and sectioning, the glandular epithelium takes up approx. 70% of the leg volume. an antenna, ca calyx, cu cuticle, fcp forcipule, fe femur, gc venom, gland units, he hemolymphatic space, m musculature, mxp maxillar palp, md mandible, n nerve, p pore of the venom duct, sc secretory cell, ta1 tarsus 1, tep telopodal gland pore, ti tibia, tg telopodal gland, vg venom gland. Scalebars in µm

To test our hypothesis that venom glands and telopodal glandular organs in *Lithobius forficatus* are serial homologues, we used a comparative morphological approach based on a combination of scanning electron microscopy (SEM), microcomputed tomography (microCT), histology and transmission electron microscopy (TEM). Examining the last four pairs of legs (pairs 12–15) showed that the medioventral faces of the distal four podomeres (femur, tibia, tarsus 1, and tarsus 2; Fig. 1A) are associated with numerous pores of aggregated telopodal gland units [22, 24]. As the glandular aggregations are divided into distinct podomeric portions in each leg, we define each of these portions as a single telopodal glandular organ that consists of closely aggregated gland units, called telopodal gland units. Total numbers and density of telopodal gland units increase towards legs located more posteriorly: while there are about 250 pores on leg 12, each ultimate leg harbours up to 5000 complex pores (±1000, *n* = 10) (Fig. 2E, F). Histology and microCT analysis of the ultimate leg’s tibia reveal that the telopodal glandular tissue is deeply sunk into the hemolymphatic space, occupying up to 70% of the leg volume (note that the drying process prior to microCT analysis reduced the volume of the glandular tissue considerably; Fig. 2F, G). The organisation of the telopodal gland organs is strikingly similar to that of the venom gland, which is also composed of an aggregation of gland units individually connected to the cuticle via pores (Fig. 2B, C). Unlike the telopodal gland organs, however, the venom gland shows an additional level of specialisation with internalisation of the cuticle to which the venom gland units are attached (termed “calyx”) via a cuticular venom duct (Fig. 2B, C).

**Fig. 3:**
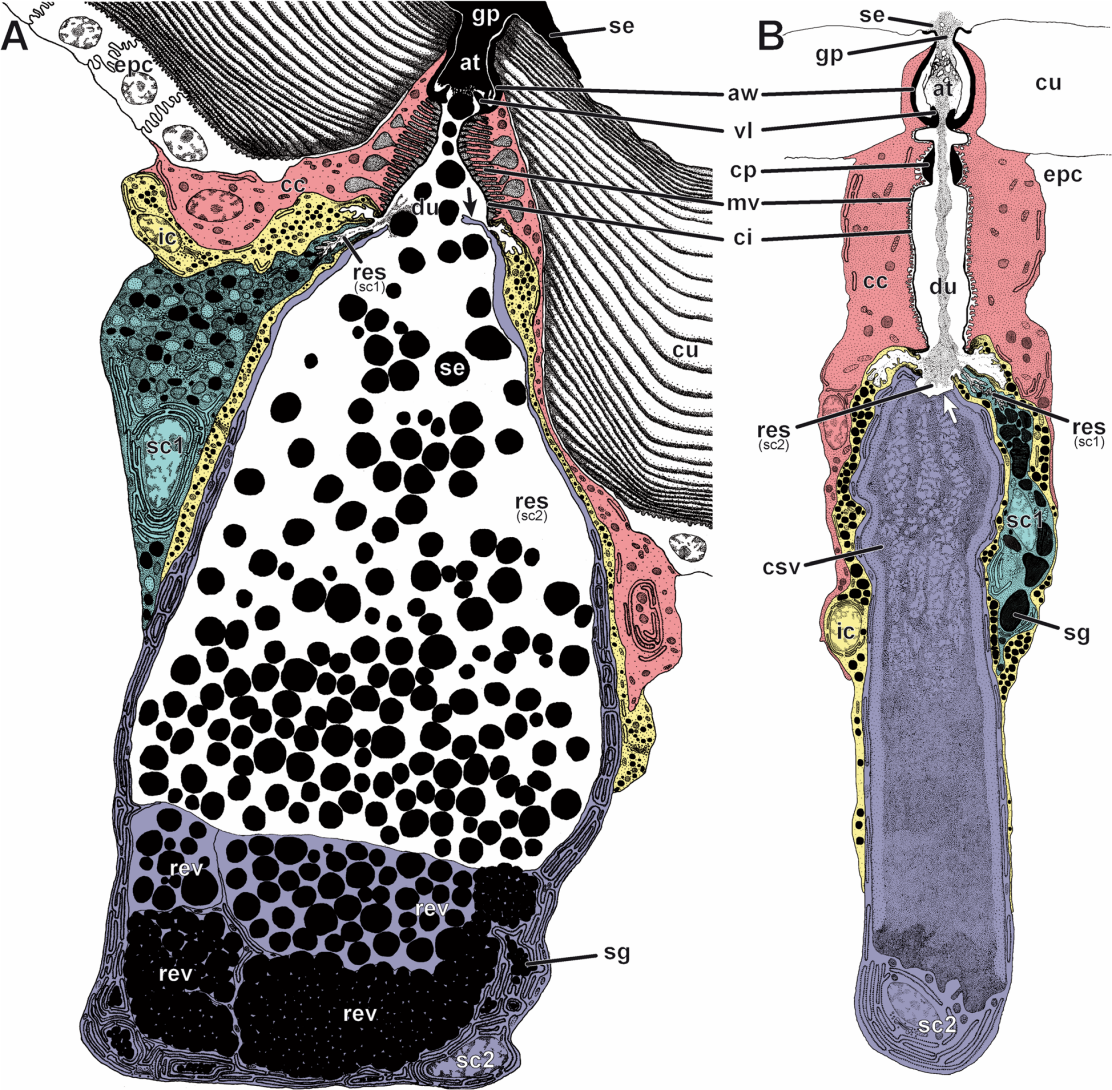
Semi-schematic reconstructions of a telopodal gland unit and a venom gland unit of *Lithobius forficatus*, analysed by TEM. **A** Telopodal gland unit and **B** venom gland unit cut along the medio-longitudinal plane. Each four-cell gland is composed of a canal cell (red), an intermediary cell (yellow) and two different types of secretory cells (sc1: turquoise, sc2: violet). The sc2 in the telopodal gland unit (**A**) is shown in active secreting state indicated by remains of the ripped apical membrane (black arrow), the content of the distalmost reservoir vacuole is released into the conducting canal, the vacuole-enclosed space becomes part of the then massively enlarged reservoir of the sc2. The venom gland unit (**B**) is drawn in dormant state indicated by the intact apical membrane of the sc2 (white arrow) separating the duct from the central (reservoir) secretory vacuole containing the secretion to be discharged. Most basal components such as the extracellular matrix, tracheae or associated parts of the nervous system are not shown in this reconstruction. Details of the lamellar system of the surrounding cuticle are also omitted in **B**. at atrium, aw atrial wall, cc canal cell, ci cuticular intima (lining the conducting canal), cp cuticular pad, csv central secretory (reservoir) vacuole, cu cuticle, cys cytoplasmic sheath of the type 2 secretory cell, du conducting canal (= duct), epc epidermal cell, ic intermediary cell, gp gland pore, mv border of microvilli formed by canal cell (connected to brush of microtubules), res reservoir, rev reservoir vacuole, sc1 type 1 secretory cell, sc2 type 2 secretory cell, sd secretion droplet, sg secretory granule, se discharged (amorphous) secretion, vl cuticular valve

Given their similar overall organisation, we next compared the architecture of the units of the venom gland and telopodal glandular organs. The external appearance, structure and diameter of the pores of telopodal gland units is nearly identical to those of solitary epidermal glands [32] that are widely dispersed across the animal's cuticle, but at about an order of magnitude lower in density (e.g. about 200 pores, ± 20, *n* = 2, on leg 10). Indeed, our TEM investigation clearly indicates that the telopodal glandular organs consist of tightly aggregated four-cell units of the recto-canal type (Fig. 3A; for classification see Müller et al. [32]). Previous descriptions of the ultrastructural organisation of the venom gland of *L. forficatus* already revealed the presence of four-cell-units, composed of two canal cells, a bulgy intermediary cell and a single secretory cell [15]. Our survey confirms the presence of aggregated four-cell units, but corrects the former study with regard to their cellular composition, namely by the finding of a single-canal cell (instead of two) but two types of secretory cells (instead of one). Rosenberg and Hilken [15] apparently mistook the type 2 secretory cell for the intermediary cell. Basically, the cellular configuration in venom gland units is highly similar to units in the telopodal glandular organs (Fig. 3B). Both share (from distal to proximal) a canal cell, an intermediary cell, two different kinds of secretory cells and appear to be innervated by neurons (for a more detailed description see also Additional file 3 Text and Figs. S1, S2) [15, 32]. The bulk of the secretion is produced by the extremely expanded type-2 secretory cell (sc2), supported by the much smaller type-1 secretory cell (sc1). Within each four-cell gland unit, the secretory cells release their secretions into the gland duct, here termed the conducting canal (du), which is formed proximally by the intermediary cell (ic) and medially and distally by the canal cell (cc). The canal is always lined by a distinct cuticular intima (ci) that is attached to a peculiar apparatus of microvilliform processes (mv) that might regulate the discharge or detention of the secretion (see discussion on extrusion mechanism by Keil [26]). The canal cell also forms the pore through the external cuticle, which consists of a valve-like structure (vl), an atrium (at) and finally the gland pore (gp).

In telopodal gland units, the secretion of the sc2 is stored in multiple, horizontally stacked reservoir vacuoles (rev), whereas only a single, tubular vacuole, called central secretory vacuole (csv), is found in venom gland units. In telopodal gland units, the distal and most voluminous reservoir opens into the conducting canal and thus represents the reservoir of the sc2 (res_sc2_) (Fig. 3A, Additional file 3 Fig. S1B). However, in venom gland units, the central secretory vacuole was always found separated from the conducting canal by a thin cytoplasmic sheet (see white arrow in Fig. 3B and Additional file 3 Fig. S2D-E). This difference of the apical membranes of the sc2 of venom and telopodal gland units is likely due to differential activity states at the time of dissection and primary fixation. Accordingly, the intact apical membrane as found in venom gland units represents the inactive state of the sc2. Content release from this reservoir vacuole probably involves ripping the very thin cytoplasmic overlay and apical membrane, as suggested by a ripped apical membrane and secretion droplets (sd) within the large reservoir (former vacuole interior) and conducting canal of the telopodal gland units (Additional file 3 Fig. S3). Such an apocrine-like mechanism of secretion (see Farkaš [33] for a review on apocrine secretion) is further supported by the absence of microvilli in the sc2 apical membrane, which is a typical feature of merocrine secretory cells in the epidermal glands of centipedes (e.g. [32]), and the presence of tubulin in the secretion (Additional file 4). In addition to similarities in organisation and ultrastructure, this observation is a major point supporting the homology of the venom gland units and telopodal gland units. Apocrine secretion in myriapods has so far only been demonstrated in the midgut epithelial cells of some millipedes and in the centipede *Scolopendra cingulata*, where it occurs via shedding of a smooth (absence of microvilli), bulb-shaped evagination of the apical cytoplasm [34–36].

Recto-canal epidermal glands containing the same setup of four cells, among them two unequally sized secretory cells, are frequently found in the epidermis of the head and appendages of *Lithobius forficatus* [32]. However, these solitary epidermal glands are much smaller in size and their sc2 is neither as tubular nor as stretched as those observed in telopodal and venom gland units. Nevertheless, we consider them homologous with regard to their specific configuration, spatial coherence and extrusion mechanism (e.g. merocrine sc1, apocrine sc2).

#### The telopodal defensive secretion is venom-like

**Fig. 4:**
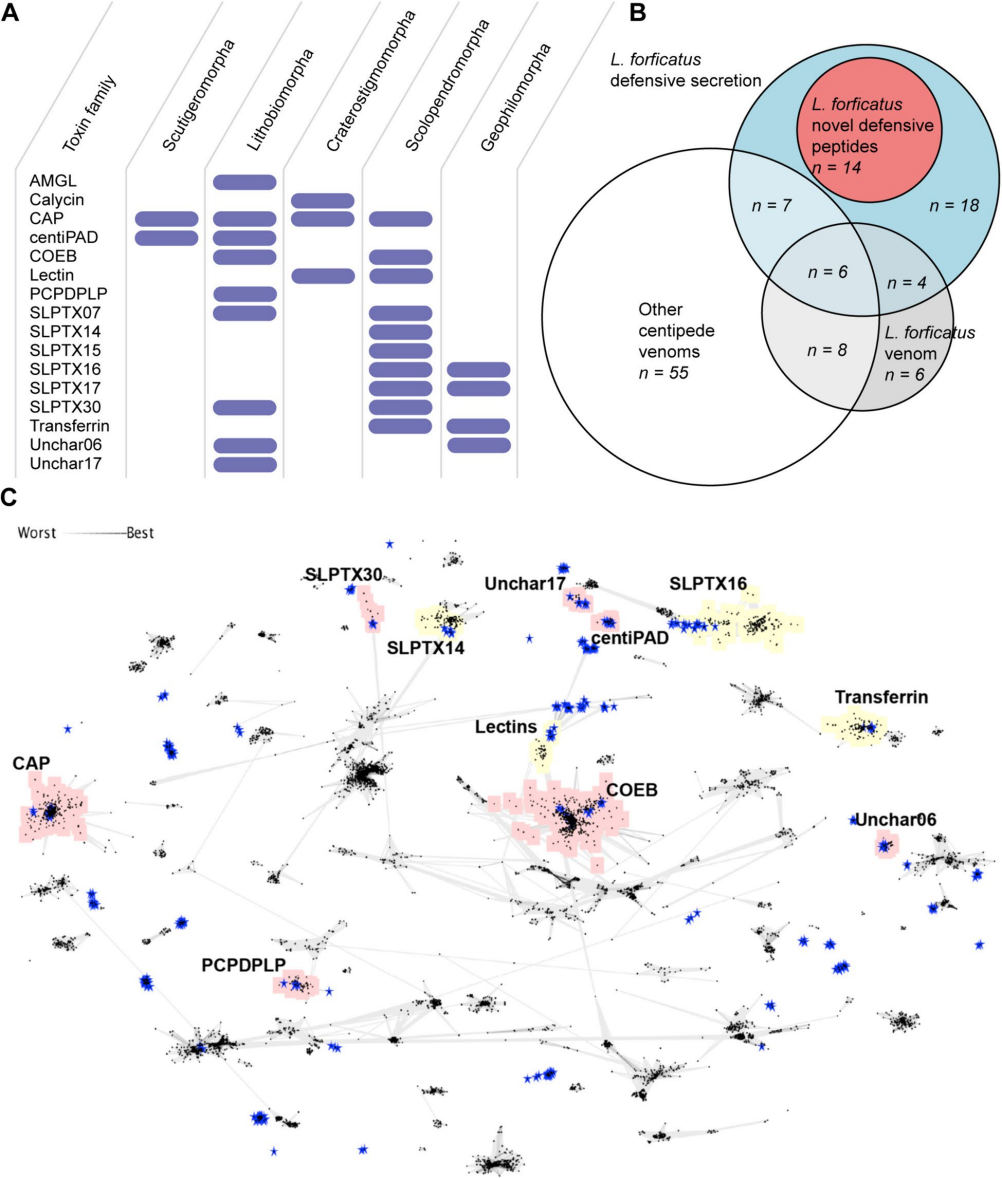
The telopodal defensive secretion shares compositional similarities with centipede venom. **A** 16 of the protein and peptide families identified in the defensive secretion are also found in the venoms of centipedes. Dark blue bars indicate previously identified venom components. **B** Total compositional overlap between protein and peptide families from all centipede venoms except *L. forficatus* venom, and *L. forficatus* telopodal defensive secretion, with the proportion of unidentifiable families from the defensive secretion shown in red. **C** Pairwise blastp-based clustering analysis of all centipede venom components (black dots) and telopodal defensive secretion components (blue stars) shows that the defensive components cluster with members of putative centipede venom toxin families. Families previously described from *L. forficatus* venom are highlighted in red, while families not previously described from *L. forficatus* venom are highlighted in yellow

To investigate how similar telopodal defensive secretion and venom are on a molecular level, we performed proteotranscriptomic analyses of the defensive secretions from two populations of *L. forficatus*: six pooled specimens from Greifswald, Germany, and three specimens from Oslo, Norway (see details in ‘Methods’). Our analysis identified 142 and 202 unique amino acid sequences from the defensive secretions of the Greifswald and Oslo populations, respectively, which we were able to classify into 27 protein and peptide families based on similarity to known protein classes (7 and 3 of which were unique to the Greifswald and Oslo population, respectively) (Additional file 4). These families include proteins with likely physiological, non-defensive roles, such as tubulin and pentraxin-like proteins, which probably reflect the apocrine-like mechanism of secretion. However, the defensive secretion is also strikingly venom-like, with 16 protein and peptide families having previously been described from centipede venoms (Fig. 4A), and many of the telopodal sequences clustering with centipede venom sequences in sequence space (Fig. 4B) [37, 38]. It is also worth noting that venoms often show substantial compositional geographic variation [39–41], although we did not look further into the cause of the regional differences in the telopodal defensive secretions of *L. forficatus*.

To be able to directly compare the venom and defensive secretion from the same specimens, we also performed a proteotranscriptomic analysis of the venom from the Oslo population, which confirmed the compositional overlap, on a protein family level, between the defensive secretion and venom. A Basic Local Alignment Search Tool (BLAST)-based clustering analysis further underscored the venom-like properties of the telopodal defensive secretion, with many of the amino acid sequences of the defensive secretion clustering tightly with components of centipede venoms (Fig. 4C).

In addition to compositional convergence with centipede venoms, many of the protein and peptide families identified in the telopodal defensive secretion also have a wide distribution across animal venoms. For example, lipocalins are found in the venoms of heteropterans [42, 43] and snakes [44], as well as in the salivary gland of ticks [45]. Epidermal growth factor (EGF) domain-containing peptides and proteins have been reported in the venom of spiders [46], ants [47–49], snakes [44, 50], cone snails [51], sea anemones [52, 53] and bloodworms [54], while general odorant-binding proteins (GOBPs) have been reported in the venoms of assassin bugs [43], wasps [55–60], ants [61, 62] and bees [63]. Additionally, lectins, including C-type lectins, can also be found expressed in other animals’ offensive and defensive fluids. Lectins are thought to play an important role in lending adhesive properties to the defensive glue of a terrestrial slug [64, 65], the Cuvierian tubules of a sea cucumber [66] and the sticky traps of nematode-catching fungi [67]. Proline- and glycine-rich proteins are prominent components of diverse sticky secretions, including the predatory secretions of onychophorans [68, 69], the defensive secretions of insects and terrestrial crustaceans [70, 71], spider silk [72], the adhesive secretions of anurans [73] and the byssus threads of bivalves [74]. The telopodal defensive secretion contains several proteins that contain a C-type lectin domain as well as large disordered proteoglycan domains, which we therefore named lectin-PGs. Disordered protein regions are liable to engage in promiscuous interactions with other molecules [75]. However, while such interactions could have adverse effects on homeostasis, they may facilitate binding of the telopodal secretions to a wide variety of different surfaces, thereby ensuring their efficacy against a broad range of potential predators.

About half of the sequences found in the telopodal defensive secretion (18 putative families) could not be matched to any known protein/peptide classes by either BLAST or InterPro searches (“Glue1–18” in Additional file 4). The majority of these (14 families) contained predicted signal peptide regions, suggesting they are indeed functional components of the defensive secretion. Among these unknown sequences were several cysteine-rich peptides (see Additional file 4), the high intramolecular connectivity of which is considered one of the hallmarks of animal venom peptide toxins [76].

#### The telopodal defensive secretion is compositionally distinct from locomotory leg solitary epidermal gland secretion

While the telopodal glandular organs appear to be functionally specialised, the secretion could still largely reflect the composition of secretions from non-specialised, solitary epidermal glands located on locomotory legs. We therefore examined the compositional overlap between the telopodal defensive secretion and non-specialised epidermal leg secretions. However, because we were unable to directly collect secretion from locomotory legs, we instead analysed the total protein contents of front legs and ultimate legs from six pooled specimens from Greifswald—obtained after TRIzol extraction of the same RNA used for transcriptomes—and compared these to each other and to the contents of the telopodal defensive secretion.

This approach revealed minimal overlap between the protein families found in the total protein content of the anterior 4 leg pairs of *L. forficatus* and those found in the telopodal defensive secretion: the 188 proteins detected in the anterior legs included only 1 of the 16 centipede venom toxin-like protein families found in the telopodal defensive secretion (transferrin, 5 amino acid sequences), none of the *L. forficatus* venom protein families, and none of the unknown telopodal peptide families (“Glue1–18”) (Additional file 4, sheets 2 and 3). We also found little overlap between protein families found in the telopodal defensive secretion and the proteomes of ultimate legs that had been completely depleted of defensive secretion 3 days prior: 9 sequences among the 194 detected proteins belonged to venom toxin-like families (C-type lectin, SLPTX16, SLPTX30), but only 1 of these families (SLPTX30) is known from *L. forficatus* venom (Additional file 4, sheets 2 and 4). These results suggest that the defensive secretion is not just regular epidermal leg secretion produced in great abundance, but is indeed a highly specialised secretion produced by functionally diverged epidermal glands.

Given that the telopodal defence systems of lithobiomorph centipedes likely evolved from locomotory legs with non-specialised, solitary recto-canal epidermal glands, we next examined how these weaponised serial homologues have diverged from those of generalised legs on a transcriptional level. We compared transcriptomic profiles for the anterior 4 pairs of locomotory legs, posterior 4 leg pairs (pairs 12–15) bearing regenerating telopodal glandular organs and forcipules bearing regenerating venom glands for each of the 3 specimens collected from the Oslo population. While this comparison includes other tissues, such as musculature, the venom glands and telopodal glandular organs occupy a large proportion of their respective appendages. However, in contrast to the low proteomic overlap between the anterior and posterior legs, differential expression analyses revealed that only 210 of 199,210 transcripts were significantly differentially expressed between the 3 sets of appendages (*P* < 0.05, at least 4-fold difference). Indeed, a principal component analysis based on all transcripts with at least five mapped fragments clustered together the transcripts of the anterior leg, posterior leg and forcipule samples from the same individuals, rather than the samples of the corresponding appendages of different individuals (Additional file 3, Fig. S4).

**Fig. 5:**
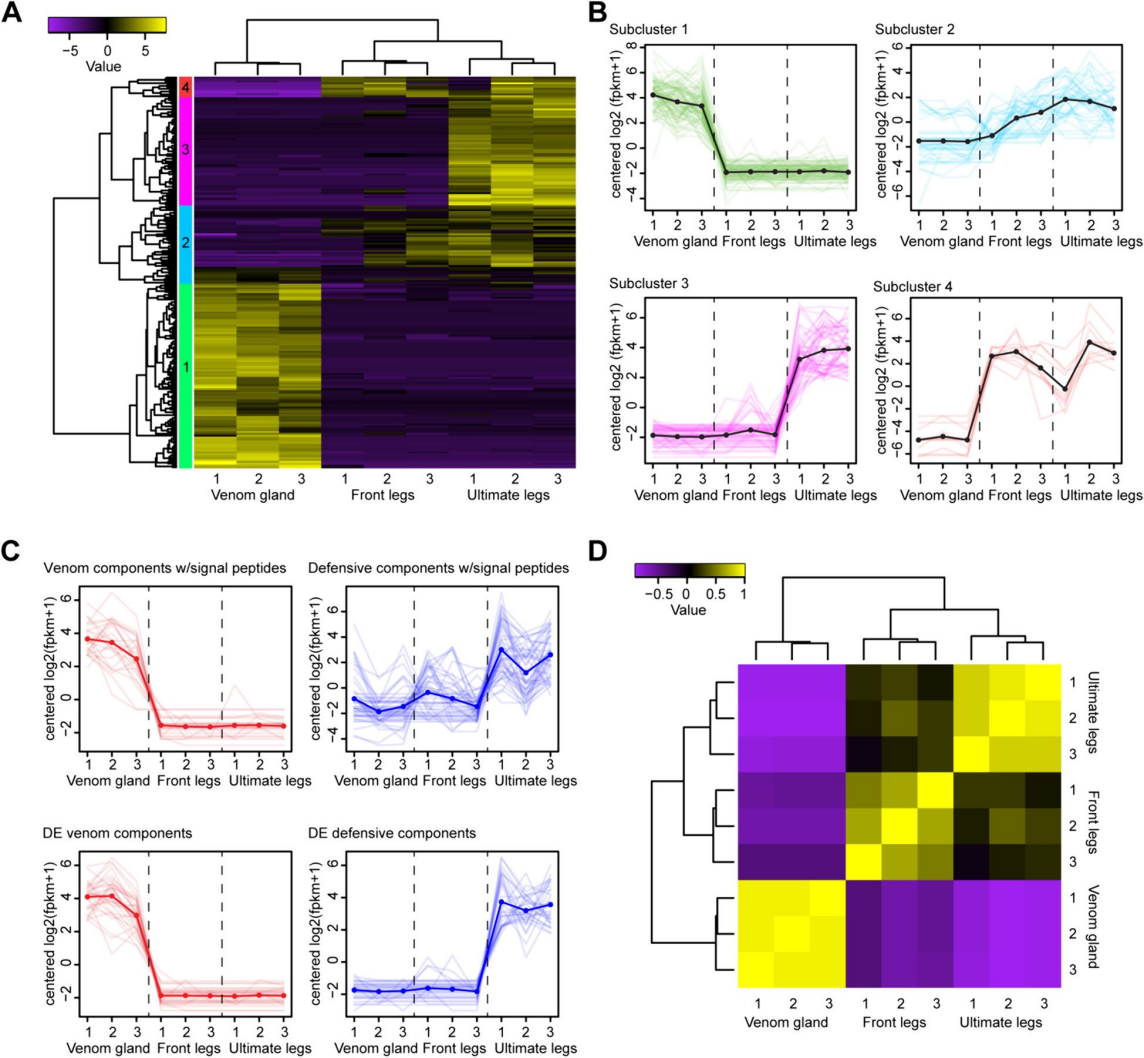
The transcriptional divergence of forcipules and ultimate legs from anterior walking legs. **A** Hierarchical clustering of differentially expressed genes (*P* < .05, C = 4) and grouping at half the length of the resulting tree yields four subclusters. Median-centred log2 FPKM expression values are shown as heatmaps and subclusters are indicated by coloured bars on the left side of the map. **B** Each subcluster shows distinct expression patterns across the forcipules, anterior walking legs and ultimate legs. The colours of lines in each graph correspond to those of the subclusters indicated in **A**. **C** The expression patterns of all venom (red) and telopodal defensive secretion (blue) components with signal peptides (top) are similar to components with significant differential expression (bottom). **D** Hierarchical clustering of samples based on correlations of differentially expressed genes show that ultimate and anterior legs are most similar, while ultimate legs and forcipules are least similar

Differentially expressed transcripts could be grouped into four subclusters (Fig. 5A) that showed distinct expression patterns (Fig. 5B). The two largest of these subclusters were significantly upregulated in either the forcipules (subcluster 1; 99 transcripts) or posterior legs (subcluster 3; 58 transcripts), while the remaining two subclusters (totalling 53 transcripts) were both generally more upregulated in the anterior and posterior legs. Although some components of the venom and—in particular—telopodal defensive secretions showed high variation in tissue expression levels between specimens, their expression tended to be higher in the appendages producing their respective secretions (Fig. 5C, top). These expression profiles also closely resembled those of the components of the venom and telopodal secretions with significant differential expression (Fig. 5C, bottom). All 80 differentially expressed venom components were upregulated in the forcipules only, 71 of 79 differentially expressed defensive secretion components were upregulated in the posterior legs only, while another 4 components were upregulated in the posterior and anterior legs compared to venom forcipules.

Strikingly, no transcripts were found to be upregulated in both forcipules and posterior legs. Indeed, hierarchical clustering analysis of differentially expressed transcripts revealed the greatest transcriptional correlation between the anterior and posterior legs and the least correlation between the posterior legs and forcipules (Fig. 5D). These results again show that the venom and telopodal defensive secretions are highly specialised and functionally converged secretions. However, the overwhelming transcriptional overlap between the three tissues also suggests the transition to functional specialisation involved only minor overall transcriptional changes in the ancestral recto-canal epidermal glands.

#### The evolution of venom and telopodal defensive secretion components

Our morphological results suggest that the ultimate legs with the telopodal glandular organs and the forcipules with the venom glands are serial homologues, a hypothesis that is boosted by the similarities of their secretions on a molecular level. Because all extant centipede lineages possess venom systems, while the telopodal defensive system has only been described for Lithobiomorpha, the venom system is thought to have evolved before the telopodal defence system. As toxins generally evolve from non-toxic body proteins—e.g. via gene duplication and neofunctionalization [77]—there are several scenarios that could explain the molecular similarities between the venom and telopodal secretions, for example: (1) toxins in the venom and telopodal secretion could have evolved independently (convergently) from the same type of non-toxic body proteins, or (2) they could have evolved from different non-toxic body proteins and acquired different toxic functions, or (3) telopodal secretion components could have been co-opted from already existing venom toxin genes, or vice versa.

To shed light on the evolution of the protein and peptide families present in the venom and telopodal defensive secretion, we examined their evolution using molecular phylogenetics. Our analyses show that each of the 16 toxin-like telopodal protein and peptide families that are present in centipede venoms (Additional file 3 Figs. S5–S18) was likely recruited into the telopodal defensive secretion only once. A previous study [37] showed that eight of the nine families that are present in both the venom and telopodal defensive secretion of *L. forficatus*—alpha-macroglobulin-like proteins, centiPAD, COEsteraseB, PCPDPLP, SLPTX07, SLPTX30, Unchar06, and Unchar17—were recruited into the venom of *L. forficatus* after the lithobiomorph lineage diverged from the other centipede lineages. Of these families, COEsteraseB includes contigs that were detected in both the venom and telopodal defensive secretion, suggesting this protein plays a dual role in these systems (Additional file 3, Fig. S5). Similarly, *L. forficatus* Unchar06 form one well-supported clade that contains both defensive and venom components, although their apparent absence from the Oslo population prevents us from directly determining whether they are present in both secretions (Additional file 3, Fig. S6).

**Fig. 6:**
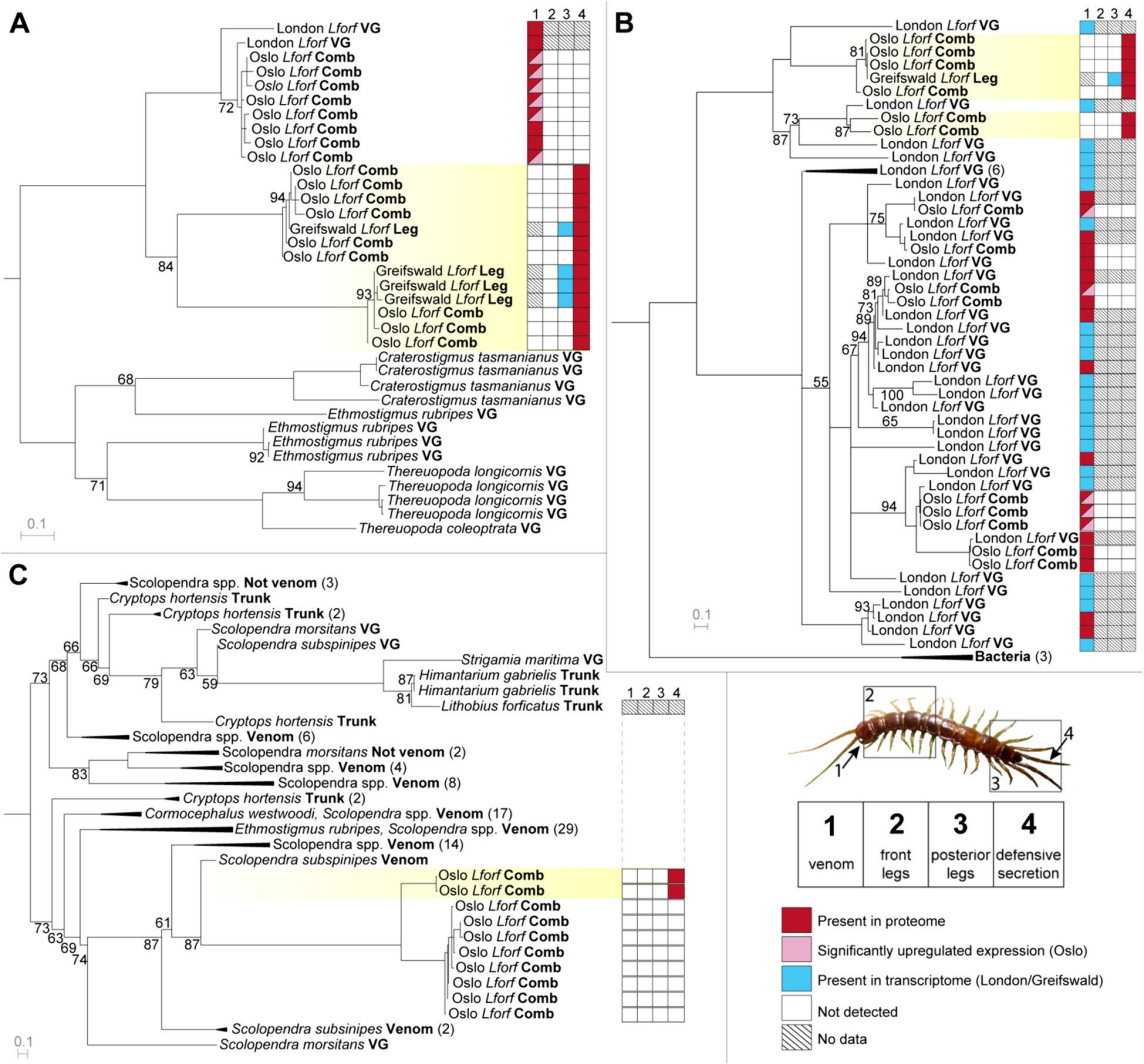
The molecular evolution of toxin-like telopodal defensive secretion components. **A** Reconstruction of the centipede CAP1 family by maximum likelihood (ML; under WAG + I + G4) shows components of the telopodal defensive secretion form two clades that probably represent separate paralogues and that are separate from the clade containing the likely ancestral *L. forficatus* venom components. **B** Reconstruction of the lithobiid PCPDPLP family by ML (under WAG + F + G4) shows that the venom and telopodal defensive secretion components form distinct clades but not whether this family was first recruited into the venom or telopodal defensive secretion. For phylogenetic tree without collapsed clades see Additional file 3, Fig. S17. **C** Reconstruction of the centipede SLPTX15 family by ML (under VT + I + I + R3) shows components of the telopodal defensive secretion do not group with the *L. forficatus* trunk sequence, but instead form a clade within venom SLPTX15 from Scolopendridae. For phylogenetic tree without collapsed clades, see Additional file 3, Fig. S18. Trees are shown as midpoint rooted, while bootstrap support values < 95 are shown at each node and nodes with support < 50 are collapsed. Sequences from this study contained in clades with identified components of the telopodal secretion are highlighted in yellow, while the presence and absence in proteomes and transcriptomes of each sequence from *L. forficatus* are indicated in the boxes behind each sequence name according to the key in the lower right panel. Tissue sources are indicated in bold for each sequence: *VG* indicates venom gland while *Comb* indicates transcriptome assembly from pooled tissues (see “Methods”). In addition, *venom* indicates sequences previously detected in venom while *Not venom* indicates sequences previously found to be part of non-venom orthogroups

The remaining protein family present in both secretions is CAP1, which was recruited into centipede venom early in their evolution [37] and was subsequently recruited into the telopodal defensive secretion. The three clades of *L. forficatus* sequences in the CAP1 tree probably correspond to different groups of paralogues, which are expressed in either the venom or the telopodal defensive secretion, but not both (Fig. 6A). Given that this family is ancestrally a venom component, our results suggest the CAP1 in the telopodal defensive secretion was co-opted from the venom CAP1.

Distinct venom and telopodal defensive secretion paralogues are probably also present in the horizontal gene transfer (HGT)-derived PCPDPLP (Fig. 6B) [78], as well as the HGT-derived centiPAD (Additional file 3, Fig. S7) [78], venom peptide families SLPTX30 (Additional file 3, Fig. S8) and SLPTX07 (Additional file 3, Fig. S9), and unknown venom protein family Unchar17 (Additional file 3, Fig. S10). Although our results do not allow us to infer the direction of recruitment, they suggest these families have diversified independently in the two secretions. In PCPDPLP, for example, there are at least two paralogues in the telopodal defensive secretion and numerous paralogues in the venom (Fig. 6B). It should be noted here that although the SLPTX07 sequences found in the venom and telopodal secretion of *L. forficatus* are very divergent, they share a conserved cysteine framework with the scolopendromorph sequences, which is a strong indication of homology in disulfide-rich peptides [76, 77]. The homology of the Unchar17 sequences expressed in the venom and telopodal glands remains somewhat uncertain because they share less than 40% sequence identity.

Of the seven centipede venom toxin-like families that were not found in the venom of *L. forficatus*, six—lectin, calycin, SLPTX14, SLPTX16, SLPTX17 and transferrin—were recruited from unknown and probably non-venom related ancestors (Additional file 3, Figs. S11–S16). In the case of SLPTX15, however, the telopodal defensive secretion components form a well-supported clade within components from the venoms of Scolopendridae (Fig. 6C) as opposed to forming a clade with non-venom orthologs expressed in the trunk (body) of *L. forficatus*. This result indicates that the telopodal defensive secretion SLPTX15 was convergently recruited from the same orthogroup as one of the main neurotoxin families in venoms of giant centipedes [79]. 

Our phylogenetic results thus show that the components of the telopodal defensive glands evolved through a combination of processes, including recruitment of non-toxin housekeeping proteins, the co-option of *L. forficatus* venom components and the recruitment of proteins not found in the venom of *L.* forficatus, but that were convergently recruited into the venoms of other centipedes. Taken together, these findings show that the evolution of the venom and telopodal defensive secretion components is a very dynamic process that does not strictly follow one direction.

## Discussion

The defensive role of the posterior and especially the ultimate legs and the telopodal glandular organs of lithobiomorph centipedes were first described by Latzel [80] and Verhoeff [29]. Panic [31] and Simon [30] showed that telopodal glandular organs are effective in predator deterrence as attackers are glued and sometimes even paralysed by filaments emanating from these glands. These intriguing observations suggest that the role of the defensive secretion is not just physical immobilisation but that it also acts as a chemical armament. Our results support the hypothesis that the defensive secretion plays a defensive role and show that the defensive secretion indeed contains a large variety of toxin-like peptides and proteins, as well as several hitherto undescribed protein types with toxin-like characteristics. The secretion is produced in numerous functionally modified four-cell units, which form dense aggregations on the last four pairs of legs, with highest abundance found on the ultimate legs. These organ-like aggregates are innervated by neurons—presumably to facilitate control over the release of the secretion—and take up the majority of the volume of the leg podomeres in which they are situated. The ultimate legs have little or no locomotory function and are instead largely dedicated to carrying out a defensive role by delivering the secretion to the attacker by either direct contact or hurling it over a distance. The defensive behaviour of the ultimate leg pair is assisted by the preceding three leg pairs (12–14), which are involved in locomotion, but less so when threatened. Thus, the ability to deploy this chemical armament depends on a series of molecular, morphological and behavioural innovations, which together form the telopodal defence system.

Forcipules with venom glands are present in all extant centipede lineages and are thought to have evolved in the last common ancestor of centipedes. In contrast, telopodal glandular organs have only been reported in the genera *Lithobius* (Lithobiidae) and *Lamyctes* (Henicopidae) [24, 26–28] and therefore probably belong to the ground pattern of Lithobiomorpha. Nevertheless, the evolutionary parallels between the telopodal defence system of the ultimate legs and the venom system of the forcipules are striking. Both systems occur in transformed trunk appendages that have specialised toxin-producing tissues, the secretions of which are used to shift antagonistic species interactions from the physical to the chemical domain. To achieve this ecological shift, both secretions evolved into distinctive cocktails of proteins with biophysical and pharmacological properties that facilitate these antagonistic interactions. The secretion components share similar evolutionary histories and are produced by functionally specialised epidermal gland units of the same four-cell type. The homology of these units is supported by several shared ultrastructural and functional traits, such as the complex pore opening through the cuticle, the compartmentalization of the conducting canal (distal atrium with valve-like closure, proximal duct compartment with fibrous microvilli-intima interface), the presence of two unequal secretory cells (type 1 and 2 cells) and the apocrine-like secretion mechanism of the type 2 secretory cells (see also Keil [26]). The only minor difference of the type 2 secretory cells in both types of glands concerns the production of a single (venom gland units) versus multiple, horizontally stacked (telopodal gland units) reservoir vacuoles.

Taken together, these results support our second hypothesis that the venom and telopodal defence systems are serially homologous traits that have convergently evolved to function as chemical weapons and that they have evolved from two sets of homomorphic traits, namely trunk appendages and solitary four-cell epidermal glands. Fascinatingly, the telopodal glandular organs represent morphologically exactly the first step in a scenario for the evolution of the centipede venom gland hypothesised by Dugon and Arthur [18], namely an aggregation of formerly solitary epidermal glands on a functionally specialised trunk appendage. The ability to inject a toxic secretion then evolved subsequently by internalising the glands and draining their secretions via a communal duct to the tip of the forcipule. As a serial homologue, the telopodal defence system thus provides indirect empirical support for the plausibility of this evolutionary scenario.

Venom glands and telopodal glandular organs may not be the only defence glands in centipedes that have evolved from solitary four-cell recto-canal epidermal glands. The geophilomorph defensive sternal glands likewise feature aggregated recto-canal epidermal glands [81–85]. In contrast to centipede venom glands and lithobiomorph telopodal glandular organs, geophilomorph sternal glands are present on trunk sternites [81–85], and their sc2 cells are significantly larger. Similar to venom glands, sternal glands are associated with musculature around the units. Indeed, the lack of architectural modification of four-cell units in venom glands, telopodal glandular organs and sternal glands may be due to a predisposition to functional innovation. It has previously been speculated that this four-cell gland type is morphologically adapted to functions requiring quick release of potentially massive amounts of secretion by having a large reservoir—the apocrine type 2 secretory cell—and bulged sub-compartments in the conducting canal, which contrasts with the slender, elongated and convoluted conducting canal in other gland types [32,83]. Our transcriptomic comparisons between the locomotory and weaponised appendages of *L. forficatus* also suggest that few modifications are required for functional innovation in these glands. Thus, the predisposition to innovation in four-cell glands appears to underlie the emergence of several novel morphological traits. It is possible that there are other defence glands in centipedes, which have evolved from the same type of four-cell gland, however, ultrastructural investigations of many centipede organ systems including glands are lacking. The evolution of epidermal glands into more complex glands has been reported for several hexapods [86] and co-option of non-specialised into functionally specialised glands appears to be a widespread phenomenon across animals. For example, the venom glands of some arthropods [42, 87–93], snakes [94], spiders [95], leeches [96, 97], cephalopods [98, 99] and even mammals [100] turned out to have evolved from salivary glands, and salt-secreting glands in tetrapods appear to have evolved from non-salt-secreting glands [101].

While there are several studies on the evolution of toxins from animal venoms [37, 102–111], and some knowledge on how glands evolved [101, 112–115], not much is known about the evolution of venom systems as a whole [116]. Our results suggest that the centipede venom system and the telopodal defence system evolved—on different levels of biological complexity—from characters that are predisposed for evolutionary innovation, i.e. recto-canal gland units (morphological level) and certain non-toxic body proteins (biomolecular level). The importance of this predisposition to evolutionary innovation is evident also among the molecular components of the defensive and venom secretions in *L. forficatus*, which share a great deal of compositional characteristics. This similarity is not surprising given the common physical requirements of the two secretions and the common evolutionary origin of their glands. However, while one might expect the secretion of an ancestral gland to be one of the main evolutionary sources of the venom and telopodal secretion, our results show that their molecular components have evolved largely through recruitment of proteins and peptides that are not expressed in the ancestral glands but are distantly related to many centipede venom toxins. This form of convergence is one of the hallmarks of the evolution of toxins in animal venoms—a phenomenon that is thought to be largely the result of physicochemical properties that make certain proteins and peptides better suited to recruitment and diversification as toxins [76, 102]. Our results suggest that these physicochemical—and probably in part functional—properties may be more important than pre-existing regulatory properties for the evolution of new functions in proteins and peptides. Together, these findings demonstrate clear parallels between molecular and morphological traits in the properties that facilitate the evolution of novelty.

## Conclusions

Taken together, our results suggest that the posterior telopodal defence system and the anterior venom system in *L. forficatus* represent serially homologous traits with convergent functions as biochemical armaments. Both glandular organs evolved via specialisation of the same type of solitary epidermal gland (four-cell recto-canal type), while their secretions evolved via convergent recruitment and co-option of proteins. The telopodal glandular organ (aggregated and specialised epidermal glands) could thus be regarded as a theoretical intermediate step in the hypothesised evolution of the venom gland (internalised gland with a duct), which occurred through gradual specialisation of existing epidermal glands. Under this scenario, the venom and telopodal gland systems have evolved from serial homologues without individuality (‘undifferentiated legs’ with identical, solitary four-cell glands) into individualised serial homologues (‘differentiated legs’ with aggregated and functionally specialized, aggregated four-cell gland units). Our results also suggest that this transition into weaponised epidermal glands and associated recruitment of physiological proteins into their biochemical arsenal was enabled by a developmental and physicochemical predisposition to evolutionary innovation. These findings further show that a continuous transformation through functional innovation of traits at lower levels of biological complexity can drive the emergence of novelties on higher levels of biological complexity.

## Methods

### Experimental animals

Adult *L. forficatus* (Linnaeus, 1758) were collected under dead wood in and around Greifswald (Germany) and Oslo (Norway) and kept individually in plastic boxes or together in a terrarium. They were provided with water and small crickets (*Acheta domesticus*) once a week. A single specimen of *Pardosa* sp. was collected on the campus of the University of Greifswald and released on the same day of the experimentation.

### Scanning and transmission electron microscopy

For SEM analysis, four male and six female adult specimens were anesthetized by cooling down in a freezer and fixed in 70% ethanol. After dissection and dehydration in a graded series of ethanol (70 to 99%), specimens were transferred to glass vials and cleaned in an ultrasonic bath. Samples were critical-point-dried using the automated dryer Leica EM CPD300 (Leica Microsystems) and mounted on copper wire (Plano #16067). For scanning electron microscopic analysis, samples were sputter-coated with gold-palladium and examined with a Zeiss EVO LS10 at the Imaging Centre of the University of Greifswald.

For TEM analysis, pieces of the ultimate legs and forcipules of four CO_2_-anaesthetized specimens were incubated in fresh Karnovsky fixative solution [83] with the aid of a Pelco BioWave Pro operated at 200 W (three pulses of microwave radiation at 2 min). After washing in several changes of phosphate buffer and 3 h of post-fixation in 2% OsO_4_ solution at room temperature, samples were dehydrated in a graded ethanol series and embedded in Epon substitute resin Embed-812 (Science Services). For (pre-) embedding protocol, see Müller et al. [117]. Ultrathin sections (55–70 nm) were prepared using a Leica UCT ultramicrotome. Serial ultrathin sections were mounted on Formvar-coated slot grids (Plano #G2500C), stained with uranyl acetate and lead citrate for 4 min each, and then examined under a ZEISS 902 A (Institute of Pathology and Neuropathology, University Hospital Essen, Germany) and a JEOL JEM-1011 (University of Greifswald, Germany) operated at 80 kV. Digital micrographs were obtained with the aid of mid-mount cameras (Morada: ZEISS 902, Essen; Megaview III, Soft Imaging System, Greifswald) using iTEM imaging software. Additional semithin sections (1 µm) were stained using 1% toluidine blue in a solution of 1% sodium tetraborate, embedded in Pertex (medite #41-4011-00) and analysed with a Nikon Eclipse 90i microscope.

#### X-ray microcomputed tomography

Two specimens were anesthetized in a freezer and fixed in Bouin’s solution overnight (cf. [118]). Preparations were washed in several changes of phosphate buffer, dehydrated in a graded ethanol series (30 to 99%) and incubated in a 1% iodine solution (iodine resublimated in 99% ethanol; Carl Roth #X864.1) for 12 h. Preparations were washed several times in pure ethanol and critical-point-dried. Finally, samples were fixed on insect pins with super glue. Scans were performed with a Zeiss Xradia MicroXCT-200 (Imaging Centre of the Department of Biology, University of Greifswald) at 40 kV, 8 W. Scan parameters for depicted male specimen were (1) 0.5 s exposure time, 4 × objective lens unit resulting in 5.087 µm pixel size; (2) 4 s exposure time, 20 × objective lens unit resulting in 0.992 µm pixel size and (3) 8 s exposure time, 40 × objective lens unit resulting in 0.456 µm pixel size. Tomography projections were reconstructed using the XMReconstructor software (Zeiss Microscopy) resulting in image stacks (TIFF format). All scans were performed using binning 2 (resulting in noise reduction) and subsequently reconstructed using binning 1 (full resolution) to avoid information loss. Volume renderings were generated using AMIRA 6.4 (ThermoFisher).

### Slow motion footage

Overview documentations of the defensive display were recorded in a small glass terrarium. Using the slowmo feature of an iPhone 6S recording at 120 frames per second, terminal trunk segments and ultimate legs of *L. forficatus* were stimulated with the tip of a brush from varying angles. For detailed documentation of an encounter of *L. forficatus* and a living opponent (*Pardosa* sp*.*), the single specimen was anesthetized using carbon dioxide and mounted on thin glass rods using PARAFILM M. A Miro LC 320S equipped with a Canon 100 mm fixed focus length objective was used to record videos at 3000 frames per second. Recordings were post-processed using Phantom Camera Control.

### Collection of venom and defensive secretions

We examined two populations of *L. forficatus* in detail: six adult *L. forficatus* (three males and three females) were collected from Greifswald, Germany—the same population as for the behavioural and morphological analyses—while three adult male *L. forficatus* were collected from Oslo, Norway. Venom was collected using electrostimulation as previously described [38] except that cooling, as opposed to CO_2_, was used to slow down specimens before immobilising them on their back using rubber bands and a clean cylindrical container. Defensive secretion was obtained from the same specimens by applying a mild electric current to the two terminal sternites and collecting the secretion with a pipette tip. Venom and defensive secretion were stored at −80 °C or below until proteomic analyses. Tissues were snap frozen in liquid nitrogen on the third day after collection (to allow RNA expression levels to rise in the replenishing glands) of telopodal defensive secretion and venom and kept at −80 °C until extraction.

### Transcriptomic analysis

For the Greifswald population, total RNA was extracted from 5 of the 6 adult *L. forficatus* used for obtaining defensive secretion (three males and two females) from leg pairs 1–4 and leg pairs 12–15 using standard TRIzol protocol (ThermoFisher, USA), pooling all front legs together and all posterior legs together. For the Oslo population, total RNA was extracted using the same protocol, except that separate extractions were performed for each of the three specimens for the forcipules, leg pairs 1–4, and leg pairs 12–15, yielding a total of nine RNA extracts. RNA samples were submitted to the University of Queensland Institute for Molecular Bioscience Sequencing Facility for library preparation and sequencing. Paired end libraries with 180 bp insert size were constructed using the Illumina TruSeq-3 Stranded mRNA kit and sequenced on an Illumina NextSeq 500 using a 300 cycle (2 × 150 bp) Mid Output Run.

For the Greifswald samples, we obtained 24,639,031 and 27,930,624 paired reads from the front and posterior legs, respectively, which were trimmed using Trimmomatic v0.35 [119] to remove adapter sequences and low-quality reads. Window function-based quality trimming was performed using a window size of 75 and a window quality of 30, and sequences with a resulting length of < 100 bp after trimming were removed, leaving 16,482,827 and 19,684,073 high-quality paired reads from the front and ultimate legs, respectively. After quality control, paired sequences from either the combined or individual libraries were assembled de novo into contigs by Trinity v2.0.6 [120] using default parameters, yielding 232,683 contigs ranging from 224 to 19,093 bp with an N50 of 781 bp. To examine the completeness of our assembly, we screened for the presence of expected near-universal single-copy orthologs from Arthropoda (arthropoda_obd10, 2020-09-10) using BUSCO v5.0.0 [121] in default transcriptome mode, returning a BUSCO score of 83.9% (single: 50.0%, duplicated: 33.9%, fragmented: 9.7%, missing: 6.4%, among 1013 searched BUSCO groups). The high level of duplication is expected because we pooled tissues from multiple specimens and did not filter our dataset prior to running the BUSCO analysis. The resulting contigs in each of the three assemblies were then translated to all possible open reading frames longer than 40 amino acids using the Galaxy tool ‘get open reading frames’ (ORFs) or coding sequences (CDSs) [122], yielding a total of 481,332 putatively translated CDSs.

For the Oslo samples, we obtained, in the order of forcipules, anterior legs and posterior legs: 10,918,732, 7,431,705 and 9,453,027 read pairs for the first specimen; 8,257,278, 2,828,623 and 8,598,039 read pairs for the second specimen; and 9,373,382, 8,079,563 and 9,272,659 read pairs for the third specimen. Trimming the raw reads with Trimmomatic v0.35, using a minimum quality of 20 across a window of 4 bases and minimum trimmed read length of 60 bp, resulted in, in the order of forcipules, anterior legs and posterior legs: 9,217,710, 6,340,183 and 8,058,339 read pairs for the first specimen; 6,941,898, 2,368,598 and 7,331,410 read pairs for the second specimen; and 7,861,103, 6,797,877 and 7,778,263 paired reads for the third specimen. All reads were then combined, and normalised and assembled de novo using Trinity v2.15.1. The resulting assembly comprised 259,676 contigs with an N50 of 1410 bp and BUSCO score of 92.9% (single: 23.3%, duplicated: 69.6%, fragmented: 4.5%, missing: 2.6%, among 1013 searched BUSCO groups from Arthropoda). We then identified and translated all putative CDSs longer than 40 amino acids using Transdecoder v5.5.0 [123], resulting in 500,606 amino acid sequences.

We used the transcriptome data from the Oslo population to examine differential expression between the three samples. To account for the high individual variation indicated by the high proportion of duplicated BUSCOs, we first clustered contig nucleotide sequences with the CD-HIT-EST function implemented in CD-HIT v4.8.1 [124], using a similarity threshold of 95% and word size of 10 bp, resulting in 199,211 contigs. We then used the ‘align_and_estimate_abundance.pl’ script of the Trinity v2.15.1 package to estimate transcript abundances in each sample, mapping the paired trimmed reads from each sample (each of the three tissue triplicate) to the non-redundant reference assembly using bowtie2 [125], and estimating abundances using RSEM v1.3.3 [126]. We then ‘used abundance_estimates_to_matrix.pl’ to assemble matrices of counts, transcripts per million (TPM) and trimmed means of M-value (TMM) normalised expression values (Additional file 6). We then used ‘PtR’ to check relationships among the sample replicates based on read counts by principal component analysis, projecting the first three PCs (--min_rowSums 5 --log2 --CPM --center_rows --prin_comp 3; Additional file 3, Fig. S4). Finally, we performed a differential expression analysis with edgeR in Bioconductor v3.15 [127–129] using ‘run_DE_analysis.pl’ and extracted differentially expressed sequences with an FDR *P*-value of 0.05 and minimum fold change of 4 using “analyze_diff_expr.pl”. To define subclusters of differentially expressed genes, we used “define_clusters_by_cutting_tree.pl”, cutting the transcript hierarchical cluster tree at half its height (--Ptree 50; Additional file 7).

### Proteomic analyses

To identify the composition of venom, telopodal defensive secretion, as well as anterior and posterior leg total protein after TRIZol RNA extraction, we analysed reduced, alkylated and trypsin digested samples by liquid chromatography–tandem mass spectrometry (LC-MS/MS). For each, 5 µg sample was reduced and alkylated by drying and re-dissolving in 4 M urea 10% acetonitrile N) 100 mM ammonium bicarbonate, pH 8. Cystines were reduced by incubating with 5 mM dithiothreitol at 70 °C for 5 min and alkylated with 10 mM iodoacetamide at 37 °C for 90 min. The reduced and alkylated samples were digested by incubating with 30 µg/µL trypsin overnight at 37 °C in 2 M urea 10% ACN 100 mM ammonium bicarbonate, pH 8, at a final substrate to enzyme ratio of approximately 100:1. The digested sample was desalted using a C18 ZipTip (ThermoFisher, USA), dried using vacuum centrifugation, dissolved in 0.5% formic acid (FA) and 2 µg of the digest analysed on an AB Sciex 5600 TripleTOF equipped with a Turbo-V source heated to 550 °C. Tryptic peptides were fractionated on a Shimadzu (Kyoto, Japan) Nexera UHPLC with an Agilent Zorbax stable-bond C18 column (Agilent, USA) (2.1 × 100 mm, 1.8 µm particle size, 300 Å pore size), using a flow rate of 180 µL/min and a gradient of 1–40% solvent B (90% ACN, 0.1% formic acid [FA]) in 0.1% FA over 60 min. MS1 spectra were acquired at 300–1800 m/z with an accumulation time of 250 ms and selecting the 20 most intense ions for MS2 scans acquired at 80–1400 m/z with an accumulation time of 100 ms and optimised for high resolution. Precursor ions with a charge of +2 to +5 and an intensity of at least 120 counts/s were selected, with a unit mass precursor ion inclusion window of ±0.7 Da, and excluding isotopes within ± 2 Da for MS/MS.

To identify proteins and peptides, we used Protein Pilot v4.4 (AB SCIEX, USA) to search the resulting MS/MS spectra against the respective translated transcriptomes from the Greifswald or Oslo populations. While biological modifications were allowed, we did not allow for amino acid substitutions in an attempt to reduce the number of false positive identifications of any similar non-toxin or non-defensive homologues. False positives were identified using decoy-based false discovery rates (FDR) as estimated by Protein Pilot, and only protein identifications with a corresponding local FDR of < 0.5% were considered significant. We then used custom workflows in Galaxy version 18.09 [130] to extract the sequences of identified toxin and non-toxin like components and used cd-hit v4.6.8-2017-1208 to cluster identical amino acid sequences.

### Annotation and phylogenetic analyses

Sequences identified proteomically were first searched against a custom database of annotated centipede toxins using blastp in blast+ v2.10.1 [131], as described previously (E-value cutoff 10^–6^) [37] (Additional file 8). Sequences showing no similarity to known centipede toxins were then searched against UniProtKB [132] using blastp with default settings and searched against InterPro v69.0 [133].

Alignments (Additional file 5) were either generated in Geneious v11.1.5 [134] using the mafft plugin [135] (Algorithm: L-INS-I, Scoring matrix: BLOSUM62, Gap open penalty: 1.53, offset value: 0.123) or using the standalone version of mafft v7.505. Annotation with InterProScan 5 [136] and blastp searches were done using plugins in Geneious or local standalone versions (versions 5.57 and 2.10, respectively). Identical protein sequences were retained in alignments if each of these corresponds to a unique transcript. Maximum likelihood trees were calculated with IQ-Tree v2.2.0 [137], using ModelFinder [138] to find the best model, and ultrafast bootstrap [139] with 10,000 replicates. Branches with bootstrap values below 50 were collapsed into multifurcations, and trees were finally visualised and shown as mid-point rooted using Archaeopteryx 0.9928 beta (180705) [140].

### Compositional comparisons

We used the jolars/eulerr R-package (https://github.com/jolars/eulerr) to generate area-proportional venn diagrams of the compositional overlap on a venom protein family level between the telopodal defensive secretion, *L. forficatus* venom, and other centipede venoms described previously [37]. The resulting venn diagram (Fig. 4B) accurately reflects the proportional overlaps of each group, with an overall a diagonal error of 2.705E-08 and stress of 9.039E-15.

To visualise the compositional overlap on a primary structural level between the *L. forficatus* telopodal defensive secretion and centipede venoms, we used CLANS [141] to cluster their components based on all-against-all pairwise blastp E-values. We first used the CLANS web-utility (https://toolkit.tuebingen.mpg.de/tools/clans) to perform an all-against-all pairwise blastp analysis, using default parameters, of all components of the telopodal defensive and venom secretions identified from the Greifswald (telopodal defensive secretion only) and Oslo (both secretions) by our proteomic analyses (Additional file 4), combined with all annotated sequences from Jenner et al. [37] (Additional file 8). We then used the Java-based CLANS tool to cluster and visualise the resulting similarity matrix of all 6029 sequences (Additional file 9), using *P*-values better than 1 and otherwise default parameters. CLANS was also used to automatically identify sequence clusters based on linkage, defined clusters as having minimum 1 link between minimum 2 sequences, yielding 72 clusters.

### Supplementary Information

**Additional file 1.** Supplementary video in mp4 format showing stereotypical defensive behaviour of *L. forficatus* against an inanimate object, 120 fps. 0:00 Weak, stereotypical response. 0:15 Strong response with jumping and shaking. 0:25 Strong response with jumping and grasping. 0:45 No response, habituation. 1:00 Weak, stereotypical response, dishabituation.

**Additional file 2.** Supplementary video in mp4 format showing defensive behaviour of *L. forficatus* against a contact with *Pardosa sp*., 3.000 fps. Ultimate legs to the front. Note 0:50 for first secretion of threads.

**Additional file 3:** Pdf containing detailed ultrastructural results and Fig. S1–15. **Fig. S1.** Ultrastructure of telopodal gland units located on the ultimate leg of *L. forficatus*. **Fig. S2.** Ultrastructure of venom gland units located in the forcipules of *L. forficatus*. **Fig. S3.** Basal aspects of telopodite gland units on the ultimate leg and venom gland units located in forcipules of *L. forficatus*. **Fig. S4.** Principal component analysis of all transcripts from anterior legs, posterior legs, and venom glands across three adult *L forficatus*. **Fig. S5.** Phylogenetic reconstructions of COEsteraseB sequences found in centipedes. **Fig. S6.** Phylogenetic reconstructions of Unchar06 sequences found in centipedes. **Fig. S7.** Phylogenetic reconstruction of centiPAD sequences found on centipedes. **Fig. S8.** Phylogenetic reconstructions of SLPTX30 sequences found in centipedes. **Fig. S9.** Phylogenetic reconstructions of SLPTX07 sequences found in centipedes. **Fig. S10.** Phylogenetic reconstructions of Unchar17 sequences found in centipedes. **Fig. S11.** Phylogenetic reconstructions of C-type lectins sequences found in centipedes. **Fig. S12.** Phylogenetic reconstructions of Calycin sequences found in centipedes. **Fig. S13.** Phylogenetic reconstructions of SLPTX14 sequences found in centipedes. **Fig. S14.** Phylogenetic reconstructions of SLPTX16 sequences found in centipedes. **Fig. S15.** Phylogenetic reconstructions of SLPTX17 sequences found in centipedes. **Fig. S16.** Phylogenetic reconstructions of Transferrin sequences found in centipedes. **Fig. S17.** Phylogenetic reconstructions of PCPDPLP sequences found in centipedes. **Fig. S18.** Phylogenetic reconstructions of SLPTX15 sequences found in centipedes.

**Additional file 4.** Excel table summarizing proteotranscriptomic results. First sheet contains results for venom and defensive secretion for the Greifswald and Oslo populations, including contigs names, protein families, presence of signal peptide region, full and predicted mature sequence, secretion containing each protein, and number of high confidence peptide matches (>95 %) in each sample. For the Oslo population, the differential expression results, and corresponding sub-cluster where applicable, are included either for each contig or for the contig retained after clustering nucleotide sequences at 95 % identity. Second sheet includes summary of results used for annotation of Greifswald data in the phylogenetic trees. Third and fourth sheets contain protein summary of the proteomic analyses of the leg proteomes of the anterior and posterior legs, respectively, trimmed to a local predicted false discovery rate < 1 %.

**Additional file 5.** Text file containing multiple sequence alignments in fasta format separated by “## <family>” that were used for phylogenetic reconstruction of toxin-like protein families identified in the telopodal secretion.

**Additional file 6.** Compressed archive with matrices of counts, transcripts per million (TPM), and trimmed means of M-value (TMM) normalised expression values.

**Additional file 7.** Compressed archive containing matrices of median-centered fpkm values for each differentially expressed sub-cluster, as well as fasta files with transcripts belonging to each sub-cluster.

**Additional file 8.** Custom centipede toxin database with sequences obtained from published datasets [37].

**Additional file 9.** All-against-all blastp similarity matrix of all 6029 sequences used in the CLANS clustering analysis.

## Data Availability

All data generated or analysed during this study are included in this published article, its supplementary information files and publicly available repositories. Transcriptomic data is available at the National Center for Biotechnology Information (NCBI) Sequence Read Archive (SRA) and Transcriptome Shotgun Assembly Sequence Database (TSA) under the BioProject ID PRJNA723641, with BioSample accessions SAMN40910206, SAMN40910207, SAMN40910208, SAMN18824803 and SAMN18824804. The Transcriptome Shotgun Assembly projects have been deposited at DDBJ/ENA/GenBank under the accessions GKTS00000000 and GKTS00000000. The versions described in this paper are the first versions, GKTS01000000 and GKTS00000000. The mass spectrometry proteomics data have been deposited to the ProteomeXchange Consortium via the PRIDE [142] partner repository with the dataset identifier PXD025512. Morphological data are available in the main text or the supplementary information. Further information and requests for resources and reagents should be directed to and will be fulfilled by the lead contacts, Eivind A. B. Undheim and Andy Sombke.

## Abbreviations

ACN: Acetonitrile
at: Four-cell unit atrium
BLAST: Basic Local Alignment Search Tool
CAP1: Cysteine-rich secretory protein, insect venom allergen antigen 5, and pathogenesis-related protein family 1
cc: Four-cell unit canal cell
CentiPAD: Centipede venom peptidyl arginine deiminase
ci: Four-cell unit cuticular intima
COEsteraseB: Carboxylesterase type B
csv: Four-cell unit central secretory vacuole
du: Four-cell unit conducting canal
EGF: Epidermal growth factor
FA: Formic acid
FDR: False discovery rates
GOBPs: General odorant-binding proteins
gp: Four-cell unit gland pore
HGT: Horizontal gene transfer
ic: Four-cell unit intermediary cell
LC-MS/MS: Liquid chromatography tandem mass spectrometry
Lectin-PG: Protein with C-type lectin domains as well as large disordered proteoglycan domains
microCT: Microcomputed tomography
mv: Four-cell unit microvilliform processes
PCPDPLP: Pesticidal crystal protein domain-containing protein-like protein
ressc2: Four-cell unit type-2 secretory cell reservoir
rev: Four-cell unit reservoir vacuoles
sc1: Four-cell unit type-1 secretory cell
sc2: Four-cell unit type-2 secretory cell
sd: Four-cell unit secretion droplets
SEM: Scanning electron microscopy
SLPTX: Scoloptoxin family, numbered
TEM: Transmission electron microscopy
Unchar6: Uncharacterised venom protein family number 6
Unchar17: Uncharacterised venom protein family number 17
vl: Four-cell unit valve-like structure
