# Supplementary material for: The venom and telopodal defence systems of the centipede Lithobius forficatus are functionally convergent serial homologues
Source: BMC Biol. 2024 Jun 13;22:135. doi: 10.1186/s12915-024-01925-x (PMC11170834; doi:10.1186/s12915-024-01925-x)
Supplement: Supplementary file 3 — Additional file 3: Pdf containing detailed ultrastructural results and Fig. S1–15. Fig. S1. Ultrastructure of telopodal gland units located on the ultimate leg of L. forficatus. Fig. S2. Ultrastructure of venom gland units located in the forcipules of L. forficatus. Fig. S3. Basal aspects of telopodite gland units on the ultimate leg and venom gland units located in forcipules of L. forficatus. Fig. S4. Principal component analysis of all transcripts from anterior legs, posterior legs, and venom glands across three adult L forficatus. Fig. S5. Phylogenetic reconstructions of COEsteraseB sequences found in centipedes. Fig. S6. Phylogenetic reconstructions of Unchar06 sequences found in centipedes. Fig. S7. Phylogenetic reconstruction of centiPAD sequences found on centipedes. Fig. S8. Phylogenetic reconstructions of SLPTX30 sequences found in centipedes. Fig. S9. Phylogenetic reconstructions of SLPTX07 sequences found in centipedes. Fig. S10. Phylogenetic reconstructions of Unchar17 sequences found in centipedes. Fig. S11. Phylogenetic reconstructions of C-type lectins sequences found in centipedes. Fig. S12. Phylogenetic reconstructions of Calycin sequences found in centipedes. Fig. S13. Phylogenetic reconstructions of SLPTX14 sequences found in centipedes. Fig. S14. Phylogenetic reconstructions of SLPTX16 sequences found in centipedes. Fig. S15. Phylogenetic reconstructions of SLPTX17 sequences found in centipedes. Fig. S16. Phylogenetic reconstructions of Transferrin sequences found in centipedes. Fig. S17. Phylogenetic reconstructions of PCPDPLP sequences found in centipedes. Fig. S18. Phylogenetic reconstructions of SLPTX15 sequences found in centipedes. [file 12915_2024_1925_MOESM3_ESM.pdf]

SUPPLEMENTARY INFORMATION

The venom and telopodal defence systems of the centipede *Lithobius forficatus* are functionally convergent serial homologues

Vanessa Schendel [1,2*], Carsten H.G. Müller [3*], Matthes Kenning [3], Michael Maxwell [1], Ronald A. Jenner [4], Eivind A. B. Undheim [1,5†], Andy Sombke [6,7†]

1 Centre for Advanced Imaging, The University of Queensland, St. Lucia, Queensland 4072, Australia
2 Institute for Molecular Bioscience, The University of Queensland, St. Lucia, Queensland 4072, Australia
3 Zoological Institute and Museum, University of Greifswald, Loitzer Strasse 26, 17489 Greifswald, Germany
4 Natural History Museum, Cromwell Road, London SW7 5BD, United Kingdom
5 Centre for Ecological and Evolutionary Synthesis, Department of Biosciences, University of Oslo, 0316 Oslo, Norway
6 Centre for Anatomy and Cell Biology, Cell and Developmental Biology, Medical University of Vienna, Schwarzspanierstrasse 17, 1090 Vienna, Austria
7 Department of Evolutionary Biology, Integrative Zoology, University of Vienna, Djerassiplatz 1, 1030 Vienna, Austria.

* Shared first authorship, † Shared last authorship, corresponding authors

Details on ultrastructural features of telopodal and venom glands

Our TEM investigation indicates that the telopodal glandular organs of *Lithobius forficatus* consist of tightly aggregated 4-cell-gland units of the recto-canal type (for classification see [32]). Each telopodal gland unit consists of (from distal to proximal) a canal cell, an intermediary cell, and two different kinds of secretory cells (**Figs. 3A, S1A**). In addition, our survey reveals that telopodal gland units are highly similar to venom gland units with regard to their general cellular architecture and many subcellular features, in particular. The venom gland shares the same pattern of tightly aggregated 4-cell-units (**Fig. 3**), but (in contrast to telopodal glands) they are deeply sunken below the epidermis (of the forcipule). In both, telopodal gland units and venom gland units, the bulk of secretion is produced by the extremely expanded, either bulgy-saccate (telopodal gland units, see **Fig. 3A**) or tubular (venom gland units, **Fig. 3B**) type-2 secretory cell (sc2). The much smaller, distinctly granulated type-1 secretory cell (sc1) adds components to this secretion. The sc1 shows an extensive system of cisternae of the rough endoplasmic reticulum as well as numerous elongated mitochondria, Golgi stacks, and many ovoid, highly electron-dense secretory droplets (sd) (**Figs. 3, S1B, S2D-E**). The apex exhibits a tiny reservoir, which is crossed by numerous microvillar processes projecting from the slightly invaginated apical membrane (**Figs. 3, S2D-E**). Sc1 and sc2 release their secretion into the gland duct, here termed as conducting canal (du), which is lined by a cuticular intima (ci) only in its most proximal part that is surrounded by the collar-like

intermediary cell (ic) (**Figs. 3, S1B,F, S2D-E**). The intermediary cell contains a moderately electron-dense cytoplasm that is rich in small, often branched mitochondria, dispersed cisternae of the rough endoplasmic reticulum, few Golgi stacks, polymorphic, extremely electron-dense granules (g), as well as a network of microtubules and filaments encompassing the apex (fm) (**Figs. 3, S1E, S2D-F**). In various places, the apical membrane is infolded to form several narrow reservoirs, which is similar to the type-1 secretory cell. However, the ic-reservoirs are invaded only by a few microvilliform processes (**Figs. 3, S2E**). The most distal part of the apical membrane of the intermediary cell is lined by the cuticular intima, which encloses the entire distal part of the duct formed by the canal cell (**Figs. 3, S1E, S2D, F)**. In both, telopodal gland units and venom gland units, the main body of the type-2 secretory cell is found at its bottom, including the ovoid nucleus and the cytoplasm, which is particularly rich in cisternae of the rough endoplasmic reticulum (rER) and secretory granules/vacuoles of various sizes (**Figs. 3, S1-2H, S3A-D**). In its medial and distal parts, however, the cytoplasm is narrowed to a thin sheath (cys_{sc2}) surrounding a single, tubular space filled with widely amorphous matter, termed central secretory vacuole (csv; only in venom gland units, see **Figs. 3B, S2D-H, S3E**) or several, often horizontally stratified compartments more or less densely filled with extremely electron-dense granules (se), termed reservoir vacuoles (rev; only in telopodal gland units, see **Figs. 3A, S1F-G, S3A-B**). Throughout the telopodal gland units investigated, the most distal reservoir vacuole was found to be continuous with the conducting canal and, consequently, represents the reservoir of the type-2 secretory cell (res) (**Figs. 3A, S1B**). In venom gland units the central amorphous space was found to be separated from the conducting canal by a thin cytoplasmic sheet and, hence, has to be considered a huge, tubular intracellular vacuole (**Figs. 3B, S2D, G-H**). Though, this ultrastructural divergence is more likely due to differential secretory activities “frozen” at the stage of dissection and primary fixation rather than being the result of different secretion mechanisms working. The median and distal part of the conducting canal is strongly compartmentalized and formed by the canal cell (cc) (**Fig. 3**). This duct region is always lined by a distinct cuticular intima (**Figs. 3, S1A-D, S2A-D**). Further traits shared by both, telopodal gland units and venom gland units concern ultrastructural details of the canal cell. These are (1) a pear-shaped distal widening of the conducting canal (the atrium (at)), which is lined by a thickened cuticular intima (the atrial wall (aw)), and penetrated by a valve-like structure (vl) (**Figs. 3, S1A, S2A-B**), and (2) a proximal duct compartment of triangular (**Fig. S1C**), elliptical or round (**Fig. S2C**) cross profile, which is lined by a thin cuticular intima. The cuticular intima of the proximal duct compartment is connected to the tips of short (venom gland units, see **Figs. 3B, S2C**) or distinct (telopodal

gland units, see **Figs. 3A, S1C-D**) microvilliform processes (mv) by brushes of filaments (fb) (see **Figs. S1C-D, S2D**). A special feature of the venom gland units is a clasp of locally thickened cuticular intima, termed 'cuticular pad' by Rosenberg and Hilken [15] present in the proximal duct compartment (**Fig. 3B**). The cytoplasm of the canal cell is generally poorly supplied with organelles. Besides some mitochondria, microtubules and rough ER cisternae, large vacuoles (va) are found close to the microvillar apparatus. The contents of these vacuoles display the same electron-density as observed in the peripheral duct space encompassed by the inner face of the cuticular intima and the microvilliform processes (**Figs. 3, S1C**).

The bottom of the sc-2 rests on a thin extracellular (basal) matrix (ecm) in both, telopodal gland units and venom gland units, which separates the glands from the subjacent or surrounding hemolymphatic space (he) (**Fig. S3**). Neurite bundles are found occasionally (telopodal gland units; see **Fig. S3B-C**) or frequently (venom gland units; see **Fig. S3E-F**) below the basal membrane of the sc-2. These neurite bundles often include neurites (neu) of various diameters as well as axonal terminations (ax), which are indicated by the presence of synaptic vesicles (syv) aggregated at and making contact to presynaptic membranes of the synaptic cleft (syc). Profiles of both regular nerve and neuroendocrine cells (nsc) can be observed (**Fig. S3C, F-G**). In addition to the extensive innervation pattern, the periphery of the venom gland is also riddled with tracheae (tr) and tracheoles reaching between strands of the multi-layered gland-associated musculature, which includes circular (cmu; outer layer), oblique (omu; median layer), and longitudinal (lmu; inner layer) muscles (e.g., **Fig. S3G**).

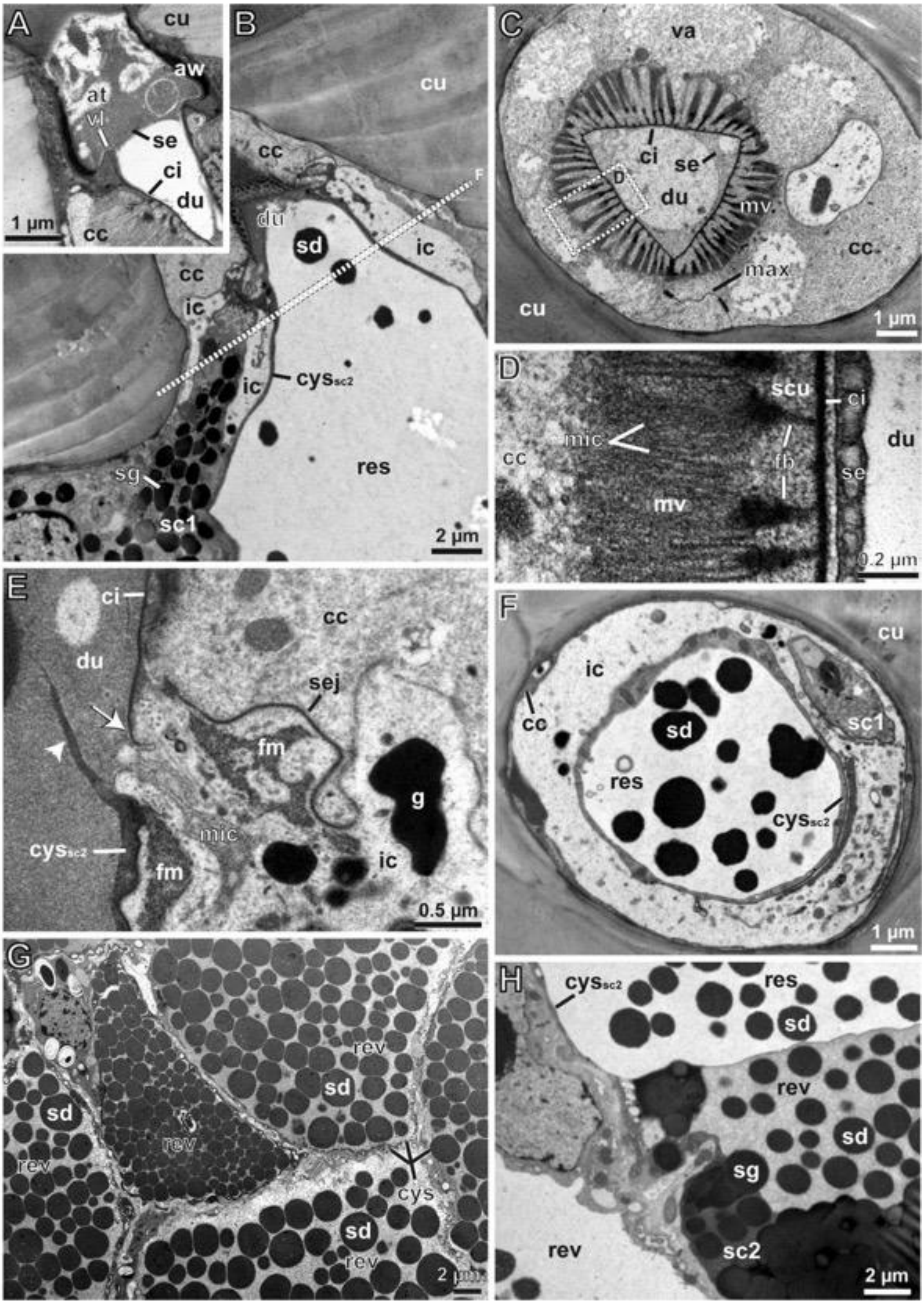

Figure S1. Ultrastructure of telopodal gland units located on the ultimate leg of *Lithobius forficatus*. Transmission electron microscopy. (**A)** Atrium filled with secretion, valve-like closing structure is not cut in full. Longitudinal section. **(B)** Longitudinal section showing the 4-cell-unit organisation: canal cell, intermediary cell, small granulated type-1 secretory cell, and large tubular type-

2 secretory cell. **(C)** Canal cell in cross-section with triangular conducting canal lined by a cuticular intima. **(D)** Detail of microvilliform processes strengthened by bundles of microtubules and attached to the cuticular intima by fibrillous brushes. Section indicated by dashed box in C. **(E)** Apex of intermediary cell in longitudinal section. Note the incomplete cuticular lining of the apical cell membrane (white arrow) and remains of the apical membrane ripped in the course of secretion discharge (white arrowhead). **(F)** Cross-section of apices of sc1 and sc2 enveloped by an 8-shaped collar of the intermediary cell. Section level indicated by dashed line in B. **(G)** Cross-section of the subcuticular portions of telopodal glands showing several type-2 secretory cells in close aggregation. Note that the huge reservoir vacuoles contain numerous droplets of secretion. The cytoplasm of the sc2 is restricted to a thin sheath around the periphery of the reservoir vacuoles. **(H)** Basal region of two sc2 cells forming a voluminous cytoplasmic sheath around bottom of reservoir vacuole.

Labels: at atrium, **aw** atrial wall cuticle, **cc** canal cell, **ci** cuticular intima, **cu** cuticle, **cys_{sc2}** cytoplasmic sheath of the type-2 secretory cell, **du** conducting canal (=duct), **fb** fibrillous bushes, **fm** filamentous matrix, **g** polymorphic granule, **ic** intermediary cell, **max** mesaxonal membrane, **mic** microtubules, **mv** microvilliform processes, **res** reservoir, **rev** reservoir vacuole, **sc1** type-1 secretory cell, **sc2** type-2 secretory cell, **scu** subcuticle, **sd** secretion droplet, **se** secretion, **sej** septate junctions, **sg** secretory granule, **va** weakly electron-dense vacuole (within canal cell), **vl** valve-like closing structure.

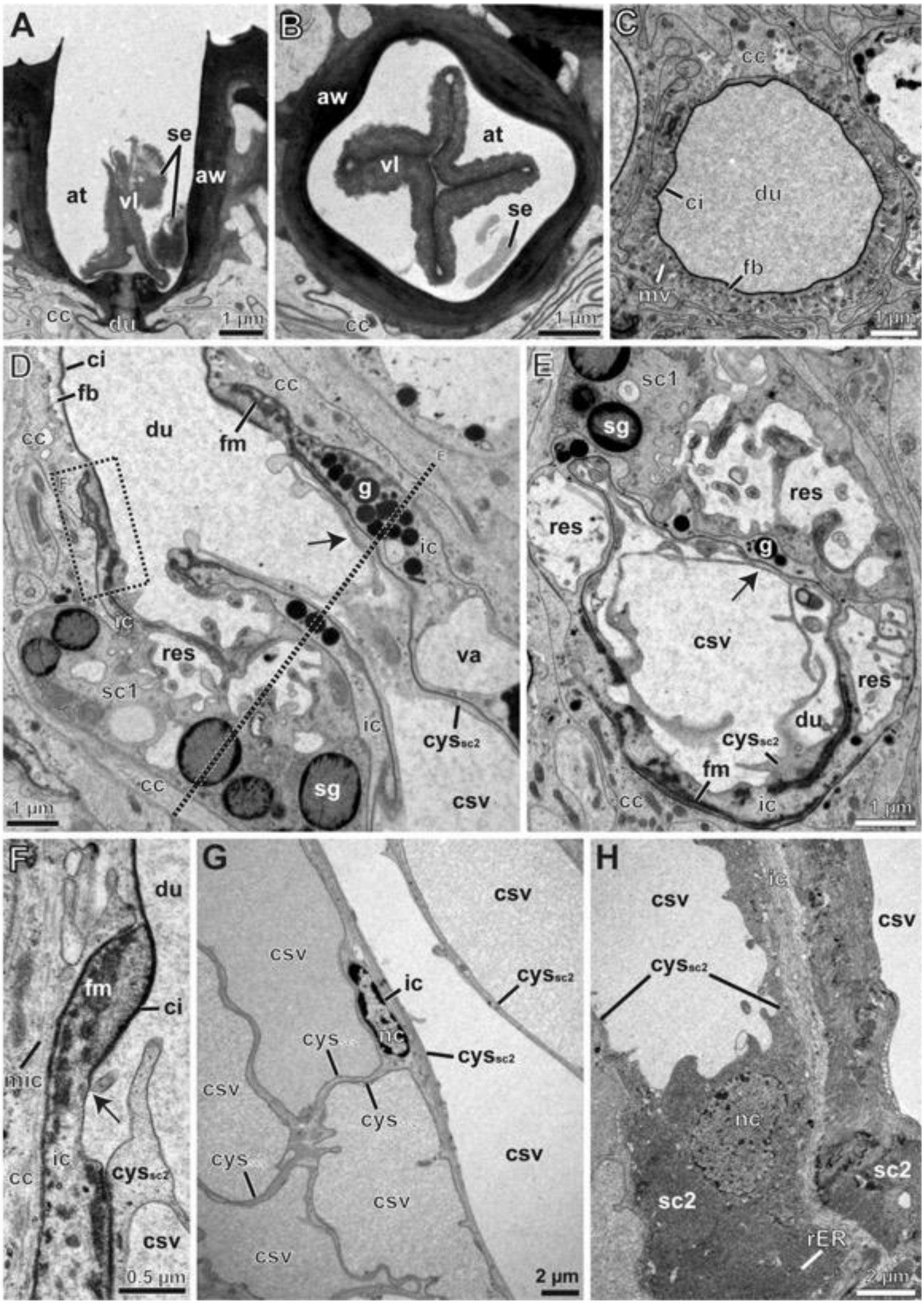

Figure S2. Ultrastructure of venom gland units located in the forcipules of *Lithobius forficatus*. Transmission electron microscopy. (**A)** Atrium equipped with a valve-like closing structure and including remains of secretion. Longitudinal section. **(B)** Atrium in cross-section with valve structure in closed state. **(C)** Median aspect of canal cell surrounding the voluminous, tubular duct lined by a

cuticular intima. Note that the apical membrane is equipped with short microvilli connected to the cuticular intima by fibrillous brushes. **(D)** Longitudinal section showing the 4-cell-unit organisation: canal cell, intermediary cell, small granulated type-1 secretory cell, and large tubular type-2 secretory cell. Note the intact apical membrane of the sc2 (black arrow) indicating pre-release state of the central secretory vacuole. **(E)** Cross-section of apices of sc1 and sc2 collared by the intermediary cell. Section level indicated by dashed line in D. Several distinct reservoirs of the intermediary cell are visible. The black arrow marks the slightly invaginated apical membrane of sc2 in cross-section. **(F)** Apex of intermediary cell in longitudinal section transition zone. The apical membrane is only lined by the cuticular intima in its distal part (black arrow). Section area indicated by dashed box in D. **(G)** Oblique-cross section through several closely adjoined venom gland units at the proximal level of the sc2, its cytoplasm is restricted to a thin sheath around the periphery of the central secretory (reservoir) vacuole. **(H)** Proximal end of two adjoined venom gland units showing the bottom aspect of the central secretory vacuole and most voluminous basal cytoplasmic region of the sc2 in longitudinal section. Both units are separated by proximal branches of the ic.

Labels: at atrium, **aw** atrial wall cuticle, **cc** canal cell, **ci** cuticular intima, **csv** central secretory vacuole, **cys**$_{\mathbf{sc2}}$ cytoplasmic sheath of the type-2 secretory cell, **du** conducting canal (=duct), **fb** fibrillous brushes, **fm** filamentous matrix, **g** electron-dense granule, **ic** intermediary cell, **mic** microtubules, **mv** microvilli, **nc** nucleus, **res** reservoir (of ic or sc1), **rER** cisternae of rough endoplasmic reticulum, **sc1** type-1 secretory cell, **sc2** type-2 secretory cell, **se** secretion, **sg** secretory granule, **va** huge, polymorphic vacuole with moderately osmiophilic content, **vl** valve-like closing structure.

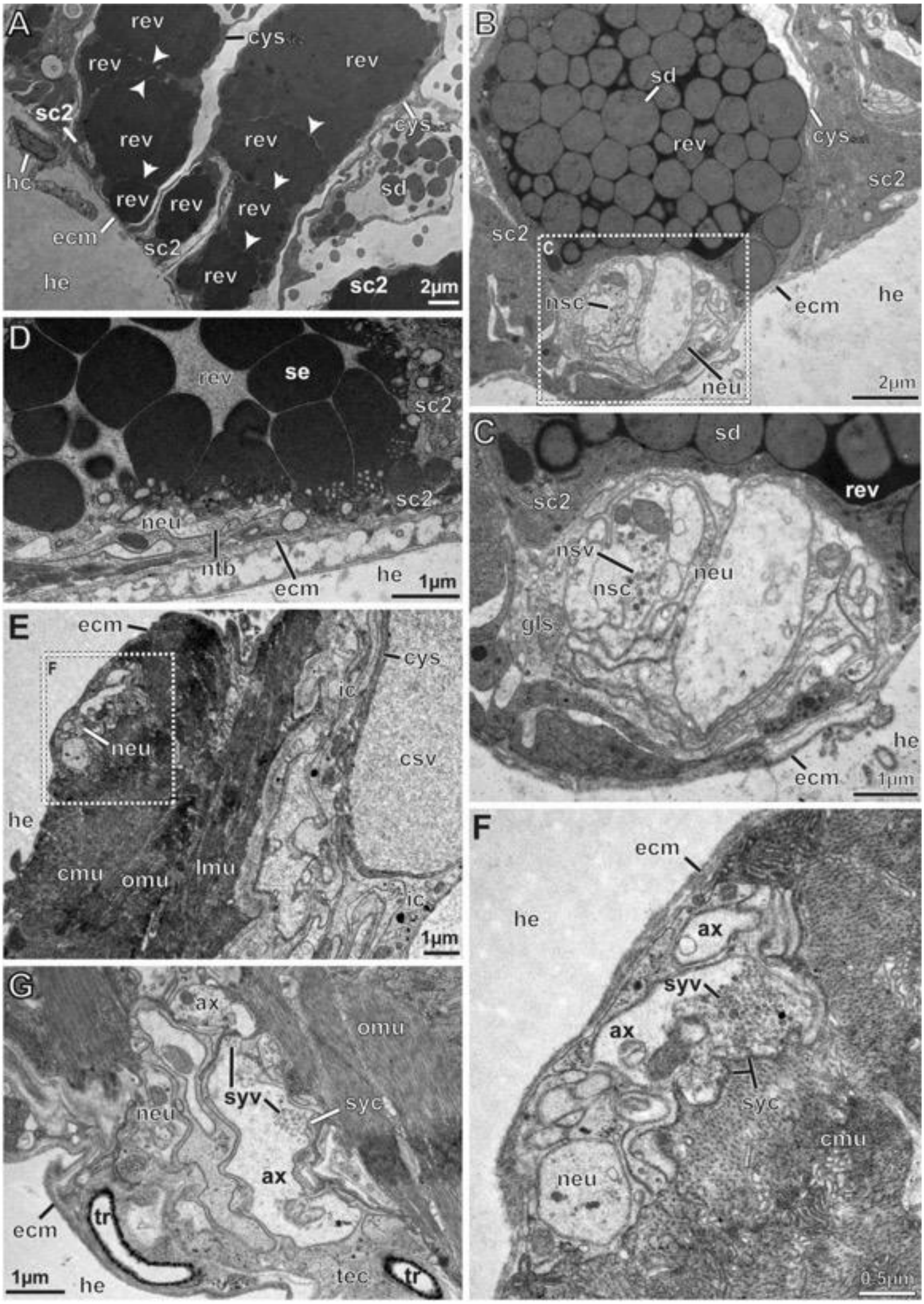

Figure S3. Basal aspects of telopodal gland units on the ultimate leg (A–D) and venom gland units located in forcipules (E–G) of *Lithobius forficatus*. Transmission electron microscopy. **(A)** Basal part of several telopodal gland units showing the bottom of type-2 secretory cells. Note the horizontally stacked system of reservoir vacuoles densely packed with secretion droplets and separated from each

other by thin cytoplasmic septa (white arrowheads). Longitudinal section. **(B)** Longitudinal section showing a single telopodal gland unit. A single reservoir vacuole is visible densely filled with secretion droplets. A distinct neurite bundle is visible below the sc2 (framed by dashed box). **(C)** Details of the neurite bundle shown in B. Neurites vary in size. A neurosecretory cell with neurosecretory vesicles indicates the efferent character of at least some of the neurites. **(D)** Longitudinal section highlighting neurite profiles, most probably axonal terminals, crammed in the interspace of the sc2 and subjacent extracellular (basal) matrix. **(E)** Longitudinal section of the mediolateral part of the venom gland showing the complex gland-associated musculature consisting of circular, oblique, and longitudinal muscles. **(F)** Magnified sector of E (see dashed box) showing a neurite bundle with several axons as indicated by numerous synaptic vesicles being in close contact to synaptic clefts. **(G)** Oblique, longitudinal section of basolateral part of the venom gland showing profiles of tracheal tubes surrounded by tracheal epithelial cells.

Labels: **ax** axon, **bl** basal labyrinth, **cmu** circular gland-associated musculature, **csv** central secretory vacuole, **cys**$_{\mathbf{sc2}}$ cytoplasmic sheath of the type-2 secretory cell, **ecm** extracellular (basal) matrix, **hc** hemocyte, **he** hemolymphatic space, **ic** intermediary cell, **lmu** longitudinal gland-associated musculature, **neu** neurite(s), **nsc** neurosecretory cell, **nsv** neurosecretory vesicles, **ntb** neurotubules, **omu** oblique gland-associated musculature, **rev** reservoir vacuole, **sc2** type-2 secretory cell, **sd** secretion droplet, **syc** synaptic cleft, **syv** synaptic vesicles, **tec** tracheal epithelial cells, **tr** tracheal tubes.

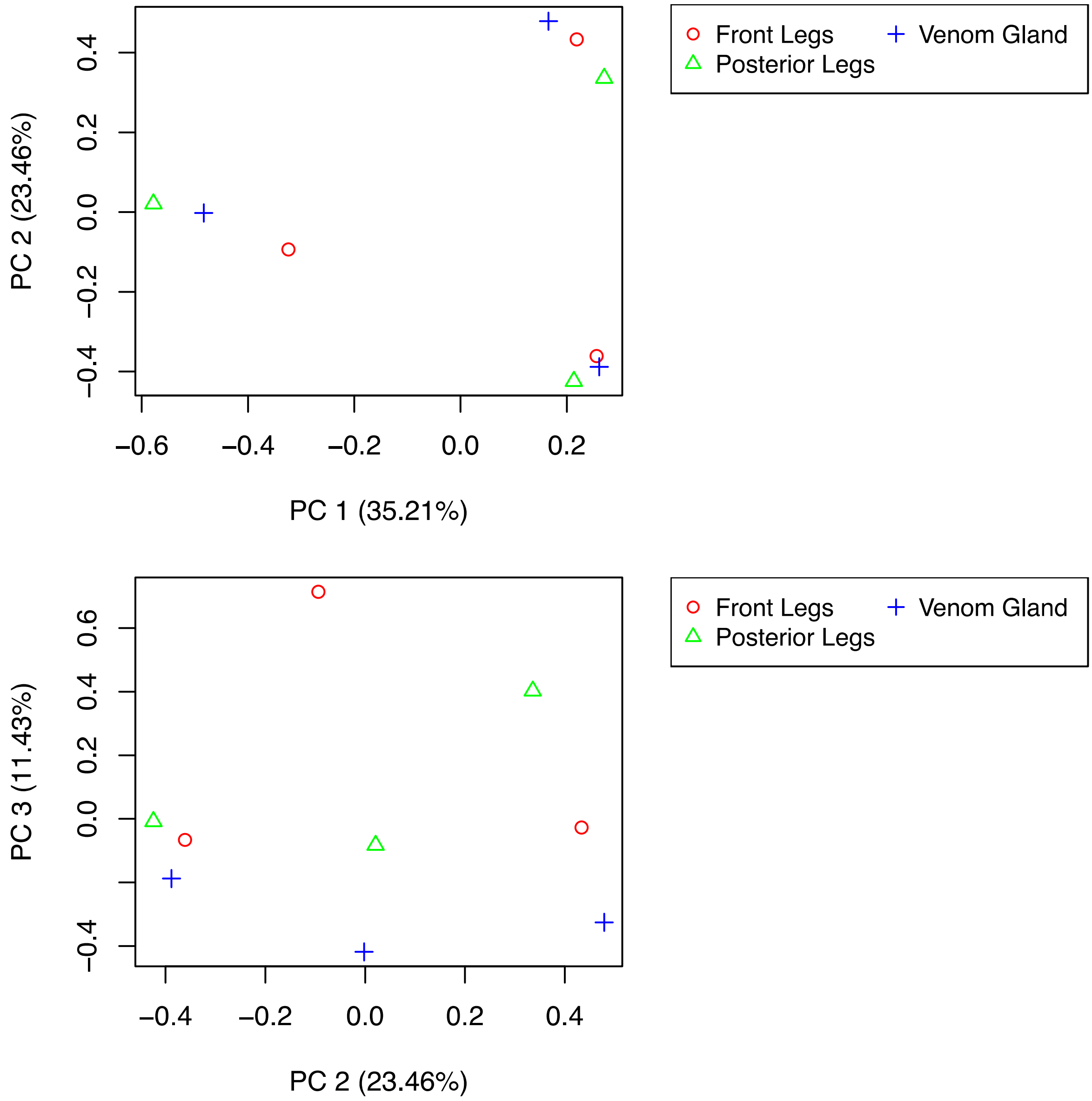

Figure S4. Principal component analysis of all transcripts from anterior legs, posterior legs, and venom glands across three adult *L forficatus*.

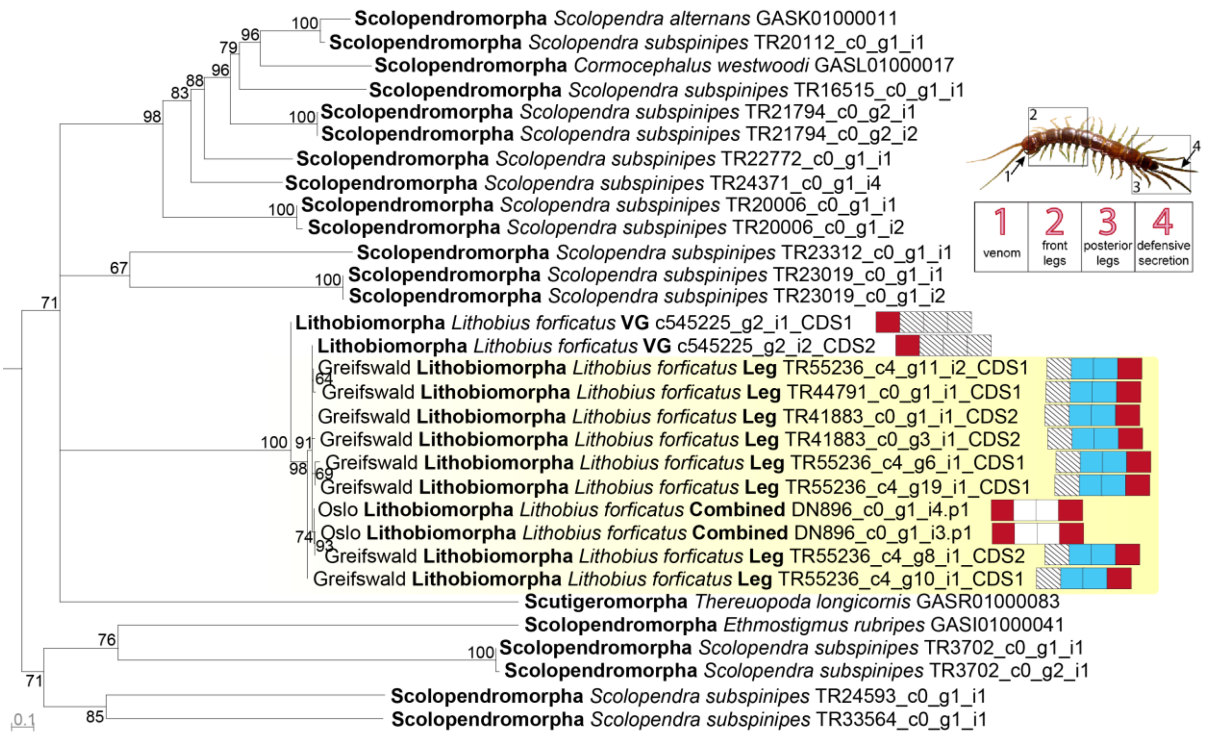

Figure S5. Phylogenetic reconstructions of COEB sequences found in centipedes. The maximum likelihood reconstructed (under LG+I+G4) consensus tree is shown as midpoint rooted, while nodes with bootstrap support values < 50 are collapsed. *L. forficatus* sequences from this study are highlighted in yellow. Boxes behind sequences indicate the sequences or absence of each in the (1) venom, (2) front legs (legs 1–4), (3) posterior legs (legs 12–15), or (4) ultimate leg telopodal secretion. Red indicates presence in proteome, blue indicates presence in transcriptome (London and Greifswald populations), pink indicates overexpression (Oslo population), while shaded indicates absence of data.

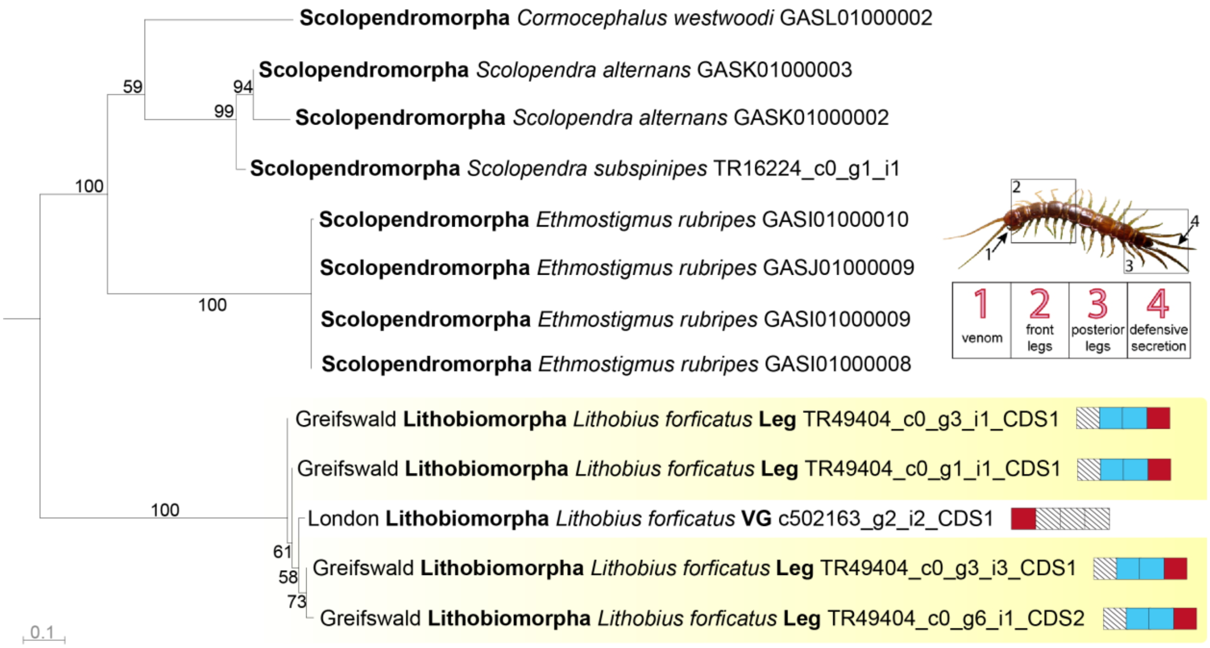

Figure S6. Phylogenetic reconstructions of Unchar06 sequences found in centipedes. The maximum likelihood reconstructed (under VT) consensus tree is shown as midpoint rooted, while nodes with bootstrap support values < 50 are collapsed. *L. forficatus* sequences from this study are highlighted in yellow. Boxes behind sequences indicate the sequences or absence of each in the (1) venom, (2) front legs (legs 1–4), (3) posterior legs (legs 12–15), or (4) ultimate leg telopodal secretion. Red indicates presence in proteome, blue indicates presence in transcriptome (London and Greifswald populations), pink indicates overexpression (Oslo population), while shaded indicates absence of data.

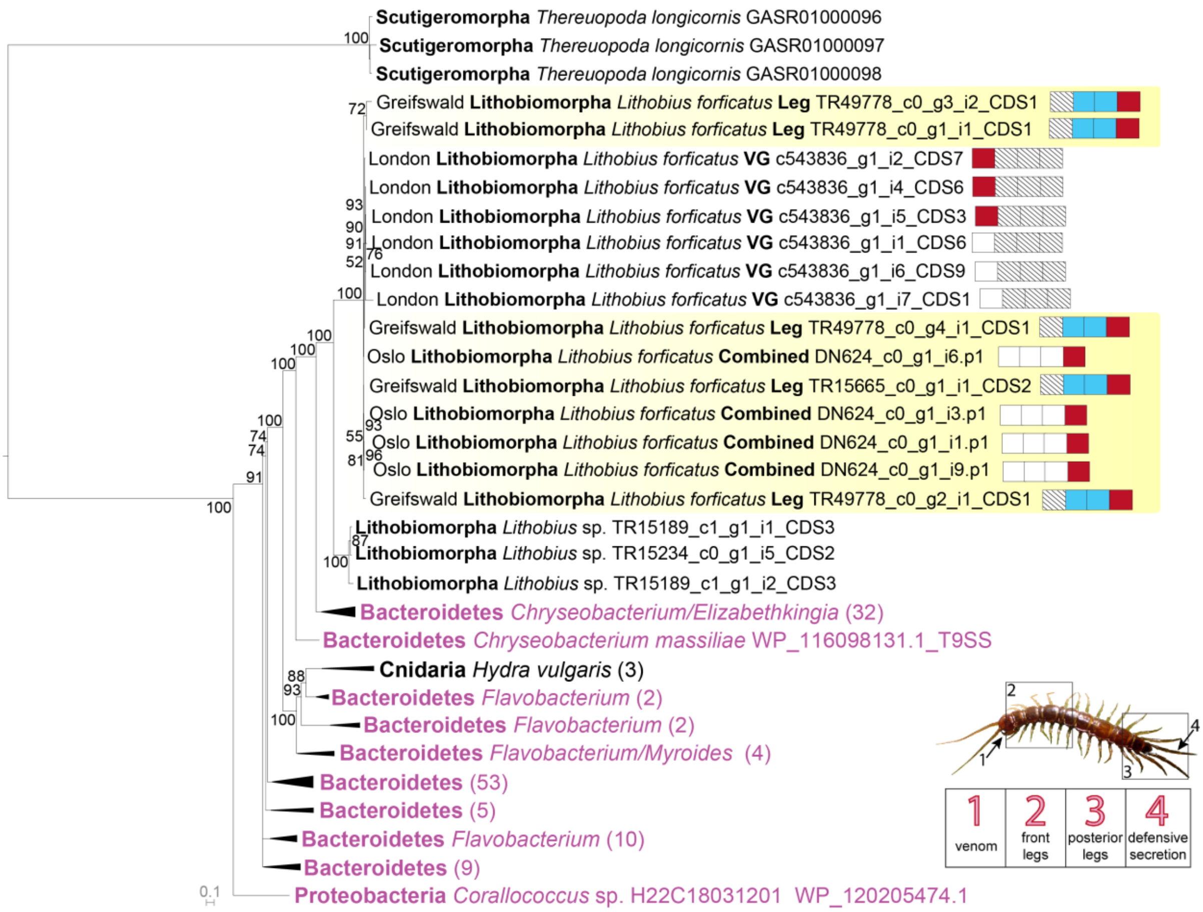

Figure S7. Phylogenetic reconstruction of centiPADs. Clades containing both venom and telopodal sequences suggest they may play dual roles in both secretions. Sequences are coloured according to their taxonomic origin, namely metazoan (black) or bacterial (pink). *L. forficatus* sequences from this study are highlighted in yellow. Boxes behind sequences indicate the sequences or absence of each in the (1) venom, (2) front legs (legs 1–4), (3) posterior legs (legs 12–15), or (4) ultimate leg telopodal secretion. Red indicates presence in proteome, blue indicates presence in transcriptome (London and Greifswald populations), pink indicates overexpression (Oslo population), while shaded indicates absence of data. The maximum likelihood reconstructed (under Q.yeast+I+R6) consensus tree is shown as midpoint rooted, while nodes with bootstrap support values < 50 are collapsed.

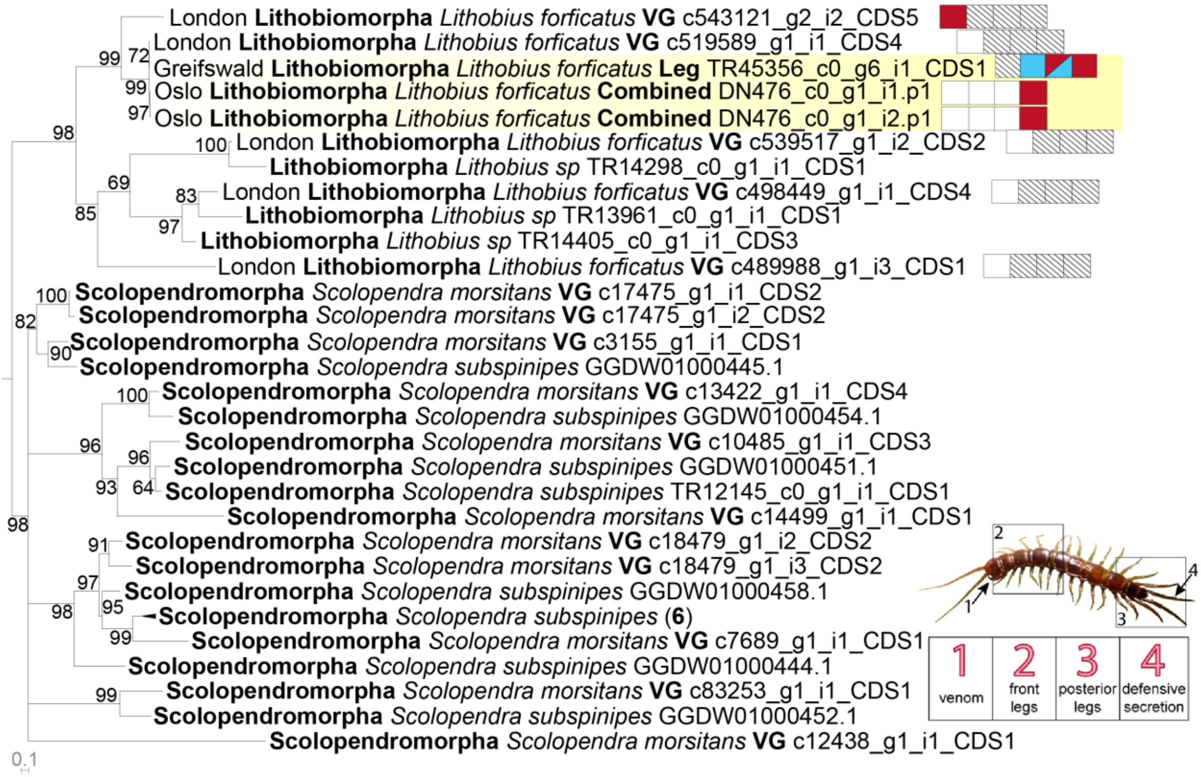

Figure S8. Phylogenetic reconstructions of SLPTX30 sequences found in centipedes. The maximum likelihood reconstructed (under LG+I+G4) consensus tree is shown as midpoint rooted, while nodes with bootstrap support values < 50 are collapsed. *L. forficatus* sequences from this study are highlighted in yellow. Boxes behind sequences indicate the sequences or absence of each in the (1) venom, (2) front legs (legs 1–4), (3) posterior legs (legs 12–15), or (4) ultimate leg telopodal secretion. Red indicates presence in proteome, blue indicates presence in transcriptome (London and Greifswald populations), pink indicates overexpression (Oslo population), while shaded indicates absence of data.

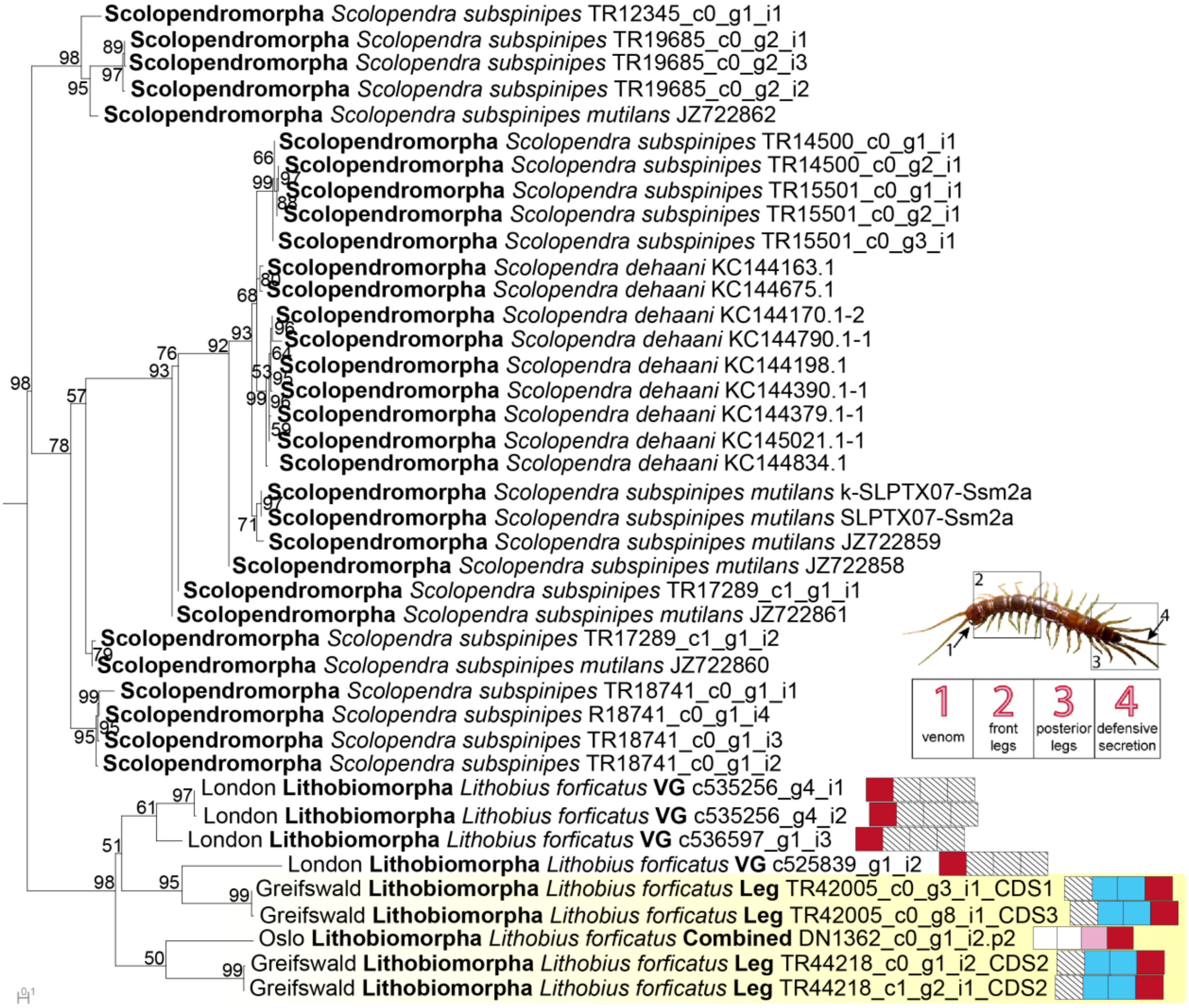

Figure S9. Phylogenetic reconstructions of SLPTX07 sequences found in centipedes. The maximum likelihood reconstructed (under WAG+I+G4) consensus tree is shown as midpoint rooted, while nodes with bootstrap support values < 50 are collapsed. *L. forficatus* sequences from this study are highlighted in yellow. Boxes behind sequences indicate the sequences or absence of each in the (1) venom, (2) front legs (legs 1–4), (3) posterior legs (legs 12–15), or (4) ultimate leg telopodal secretion. Red indicates presence in proteome, blue indicates presence in transcriptome (London and Greifswald populations), pink indicates overexpression (Oslo population), while shaded indicates absence of data.

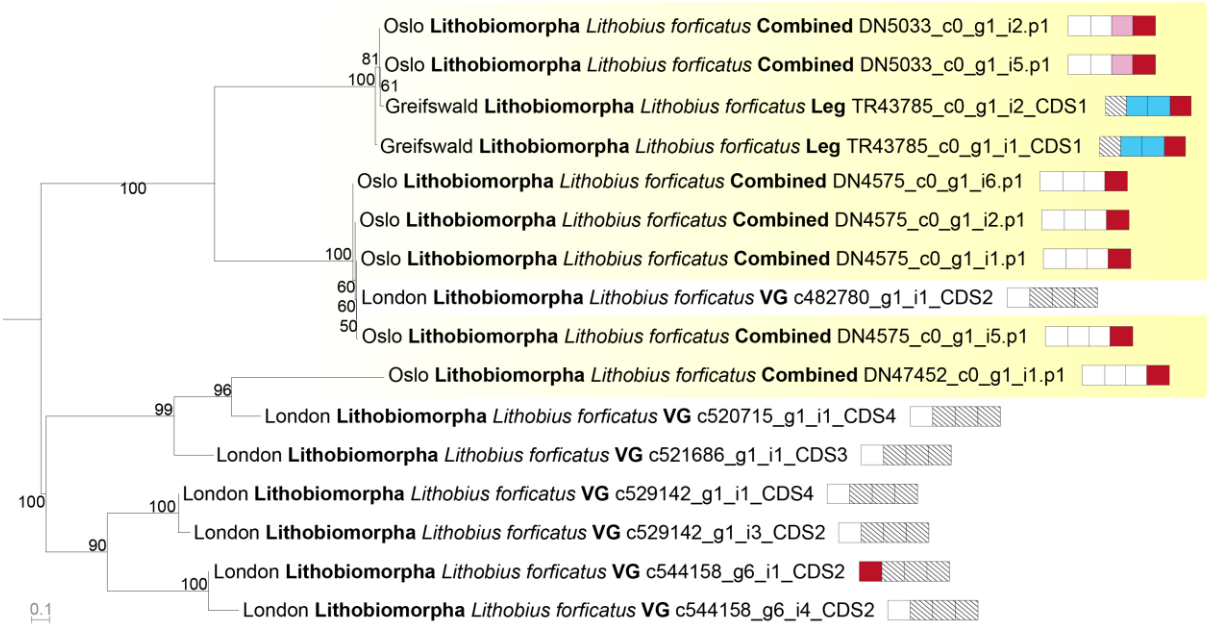

Figure S10. Phylogenetic reconstructions of Unchar17 sequences found in centipedes. The maximum likelihood reconstructed (under LG+I+G4) consensus tree is shown as midpoint rooted, while nodes with bootstrap support values < 50 are collapsed. *L. forficatus* sequences from this study are highlighted in yellow. Boxes behind sequences indicate the sequences or absence of each in the (1) venom, (2) front legs (legs 1–4), (3) posterior legs (legs 12–15), or (4) ultimate leg telopodal secretion. Red indicates presence in proteome, blue indicates presence in transcriptome (London and Greifswald populations), pink indicates overexpression (Oslo population), while shaded indicates absence of data.

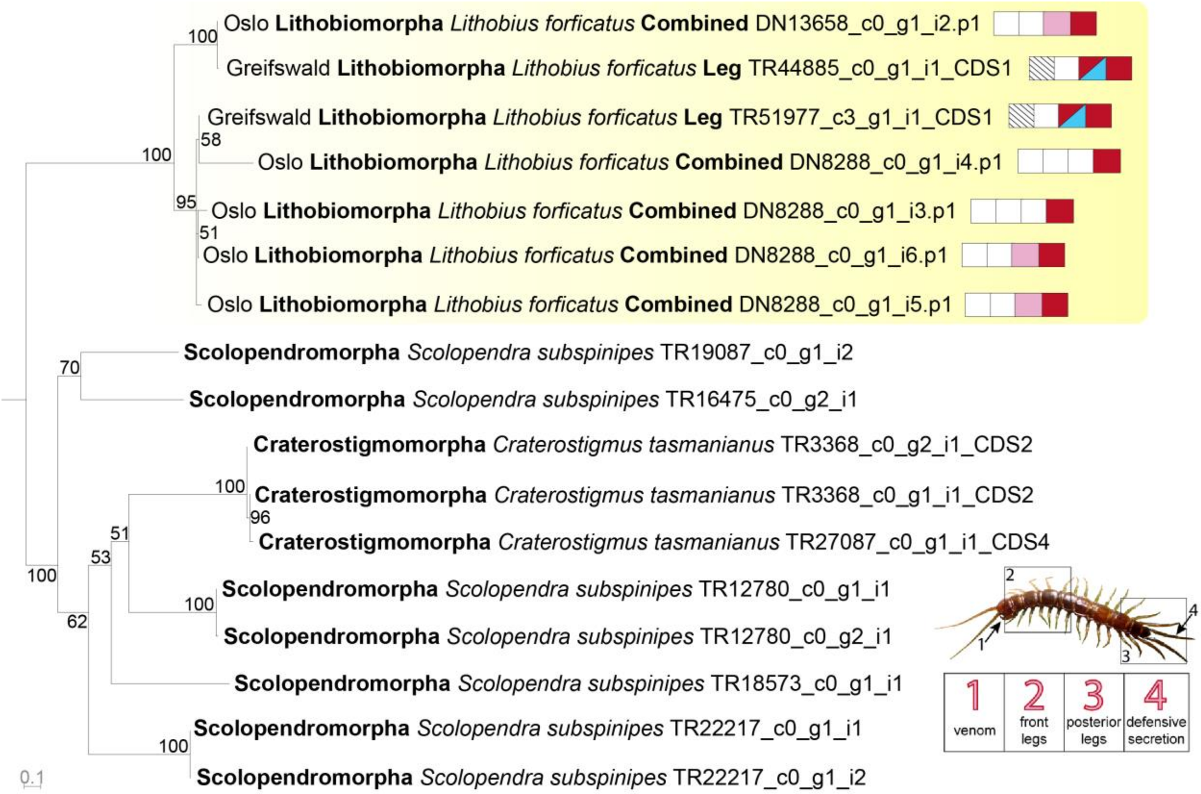

Figure S11. Phylogenetic reconstructions of C-type lectin domains from sequences found in centipedes. The maximum likelihood reconstructed (under PMB+F+G4) consensus tree is shown as midpoint rooted, while nodes with bootstrap support values < 50 are collapsed. *L. forficatus* sequences from this study are highlighted in yellow and belong to the lectin-PG family. Boxes behind sequences indicate the sequences or absence of each in the (1) venom, (2) front legs (legs 1–4), (3) posterior legs (legs 12–15), or (4) ultimate leg telopodal secretion. Red indicates presence in proteome, blue indicates presence in transcriptome (London and Greifswald populations), pink indicates overexpression (Oslo population), while shaded indicates absence of data.

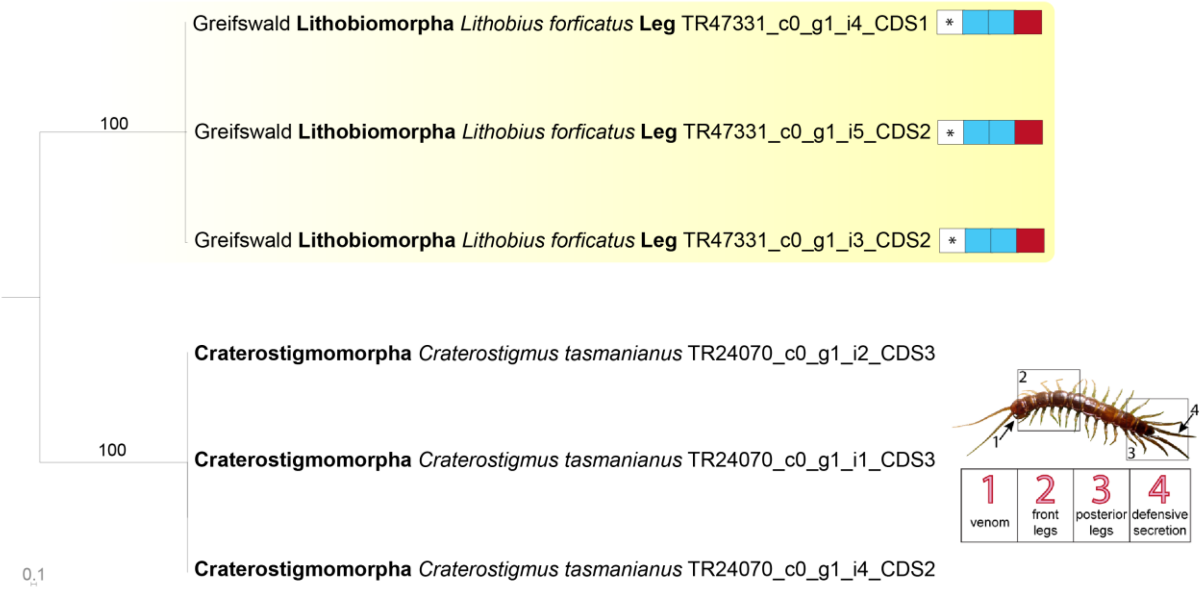

Figure S12. Phylogenetic reconstructions of Calycin sequences found in centipedes. The maximum likelihood reconstructed (under JTT+G4) consensus tree is shown as midpoint rooted, while nodes with bootstrap support values < 50 are collapsed. *L. forficatus* sequences from this study are highlighted in yellow. Boxes behind sequences indicate the sequences or absence of each in the (1) venom, (2) front legs (legs 1–4), (3) posterior legs (legs 12–15), or (4) ultimate leg telopodal secretion. Red indicates presence in proteome, blue indicates presence in transcriptome (London and Greifswald populations), pink indicates overexpression (Oslo population), while shaded indicates absence of data. Stars indicate that a highly similar sequence (95 % identity) was identified in the venom gland transcriptome, but not venom proteome, from the London population.

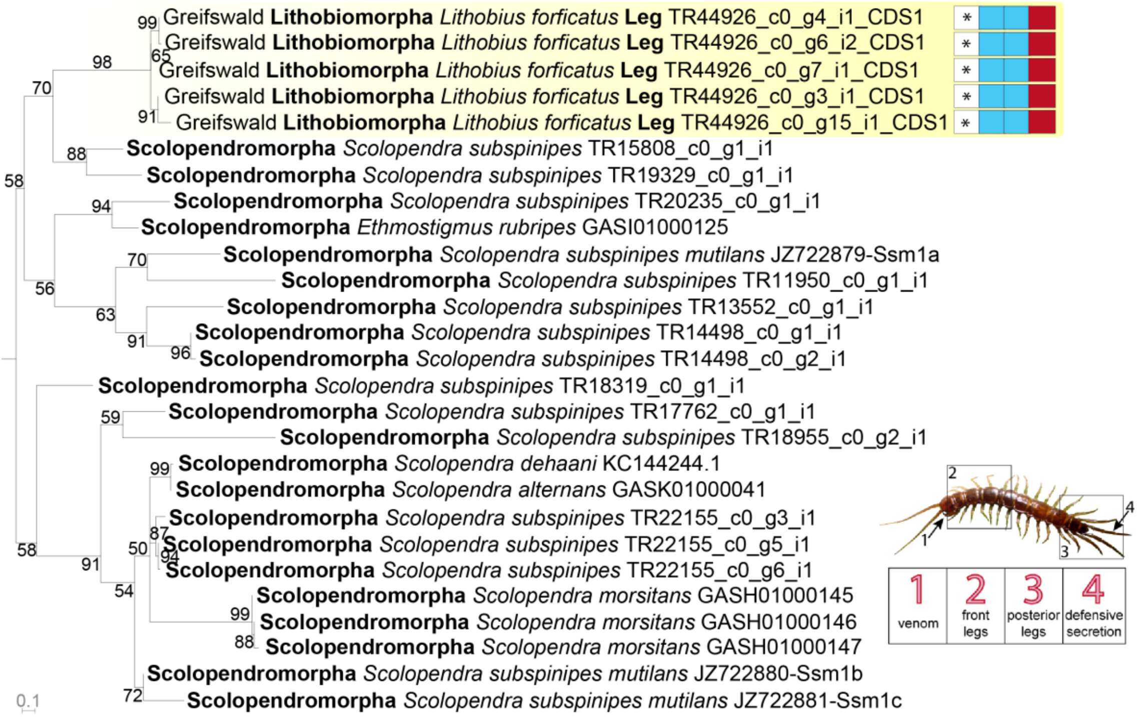

Figure S13. Phylogenetic reconstructions of SLPTX14 sequences found in centipedes. The maximum likelihood reconstructed (under VT+I+R2) consensus tree is shown as midpoint rooted, while nodes with bootstrap support values < 50 are collapsed. *L. forficatus* sequences from this study are highlighted in yellow. Boxes behind sequences indicate the sequences or absence of each in the (1) venom, (2) front legs (legs 1–4), (3) posterior legs (legs 12–15), or (4) ultimate leg telopodal secretion. Red indicates presence in proteome, blue indicates presence in transcriptome (London and Greifswald populations), pink indicates overexpression (Oslo population), while shaded indicates absence of data. Stars indicate that a highly similar sequence (95 % identity) was identified in the venom gland transcriptome, but not venom proteome, from the London population.

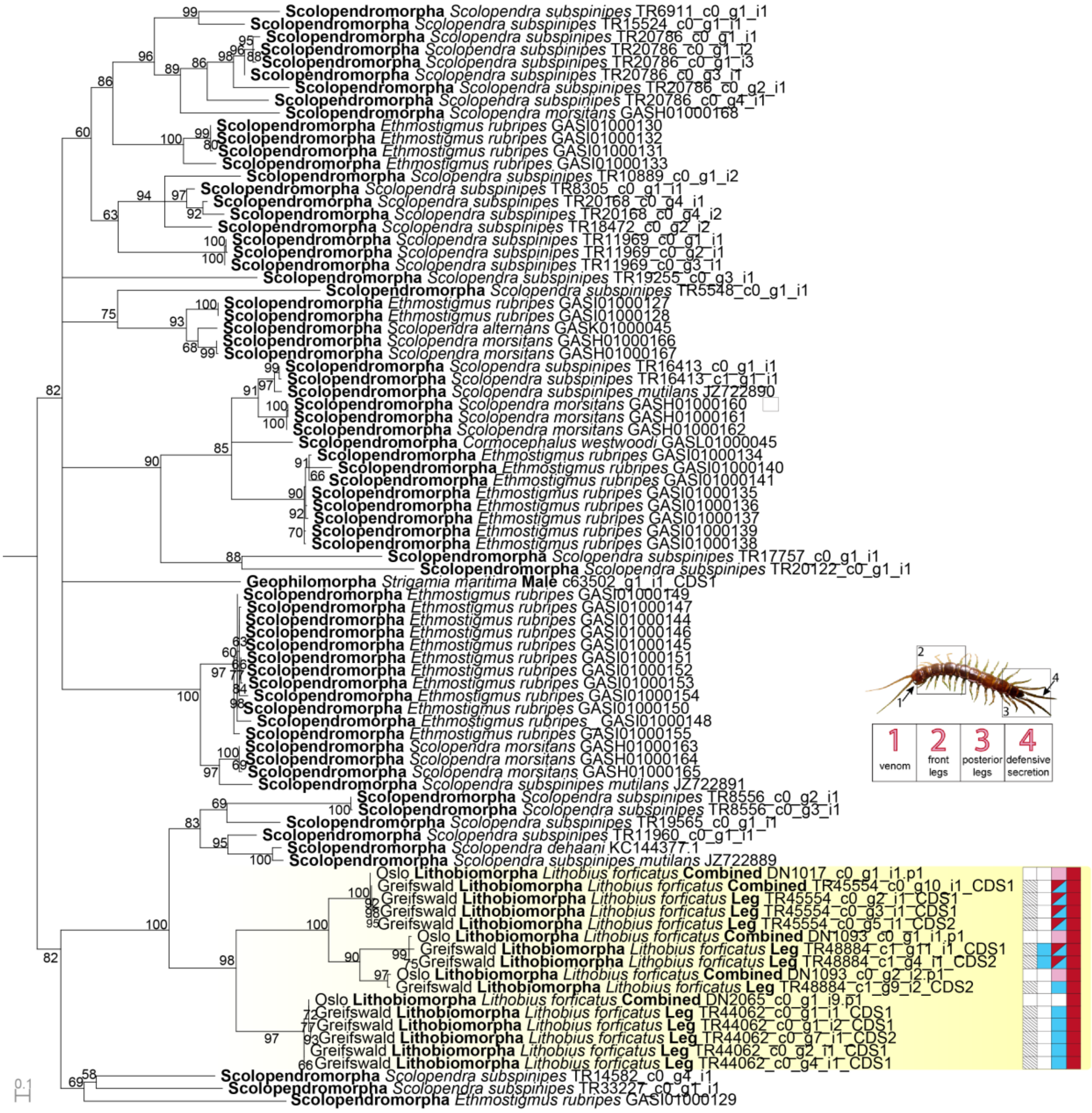

Figure S14. Phylogenetic reconstructions of SLPTX16 sequences found in centipedes. The maximum likelihood reconstructed (under VT+I+I+R3) consensus tree is shown as midpoint rooted, while nodes with bootstrap support values < 50 are collapsed. *L. forficatus* sequences from this study are highlighted in yellow. Boxes behind sequences indicate the sequences or absence of each in the (1) venom, (2) front legs (legs 1–4), (3) posterior legs (legs 12–15), or (4) ultimate leg telopodal secretion. Red indicates presence in proteome, blue indicates presence in transcriptome (London and Greifswald populations), pink indicates overexpression (Oslo population), while shaded indicates absence of data.

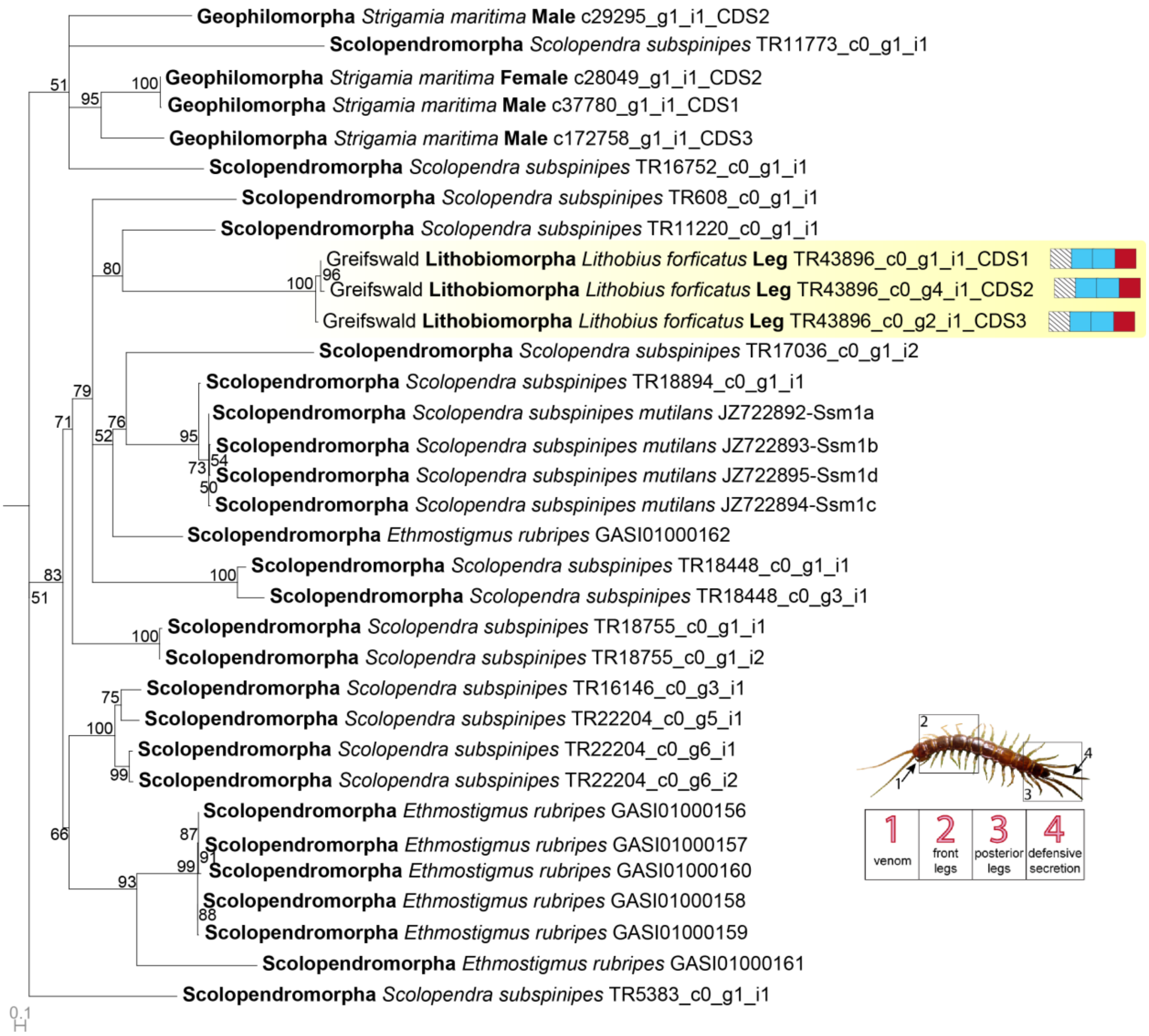

Figure S15. Phylogenetic reconstructions of SLPTX17 sequences found in centipedes. The maximum likelihood reconstructed (under LG+I+G4) consensus tree is shown as midpoint rooted, while nodes with bootstrap support values < 50 are collapsed. *L. forficatus* sequences from this study are highlighted in yellow. Boxes behind sequences indicate the sequences or absence of each in the (1) venom, (2) front legs (legs 1–4), (3) posterior legs (legs 12–15), or (4) ultimate leg telopodal secretion. Red indicates presence in proteome, blue indicates presence in transcriptome (London and Greifswald populations), pink indicates overexpression (Oslo population), while shaded indicates absence of data.

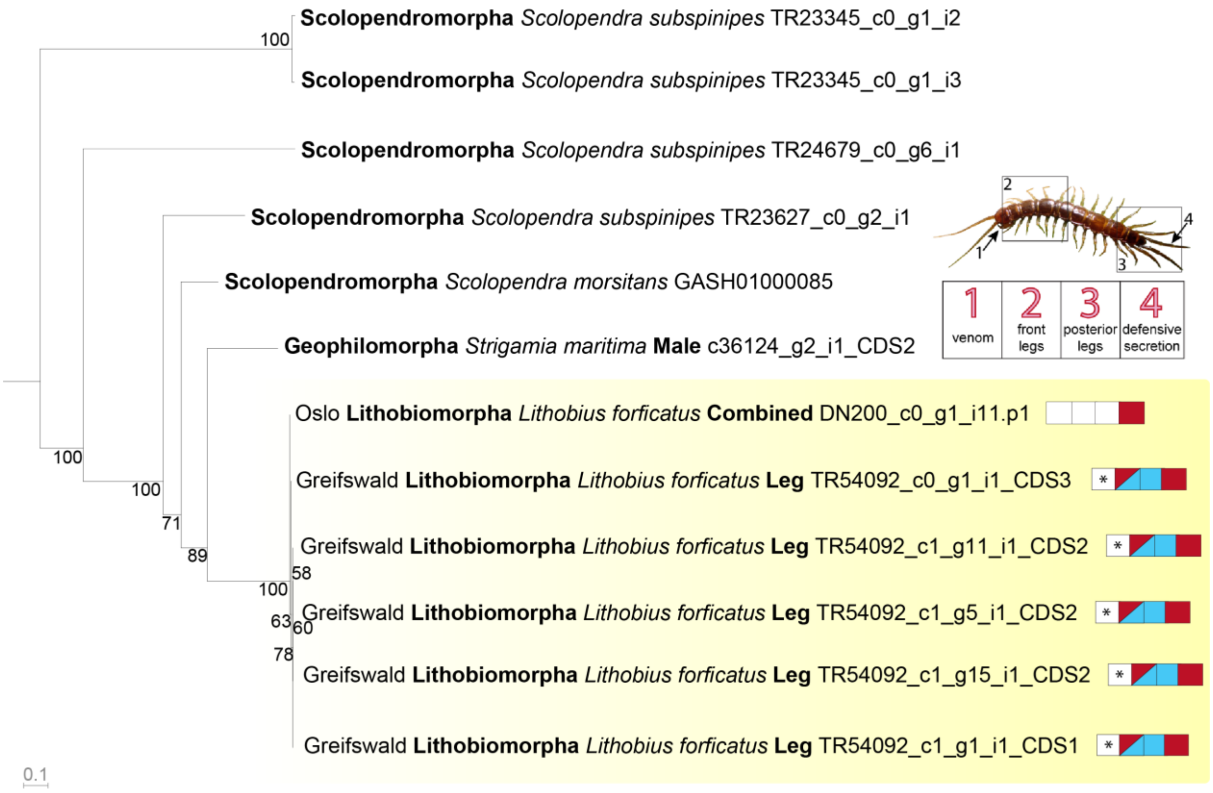

Figure S16. Phylogenetic reconstructions of Transferrin sequences found in centipedes. The maximum likelihood reconstructed (under WAG+G4) consensus tree is shown as midpoint rooted, while nodes with bootstrap support values < 50 are collapsed. *L. forficatus* sequences from this study are highlighted in yellow. Boxes behind sequences indicate the sequences or absence of each in the (1) venom, (2) front legs (legs 1–4), (3) posterior legs (legs 12–15), or (4) ultimate leg telopodal secretion. Red indicates presence in proteome, blue indicates presence in transcriptome (London and Greifswald populations), pink indicates overexpression (Oslo population), shaded indicates absence of data, while asterisk indicates that a homologue with > 90 % pairwise similarity is present in the venom gland transcriptome of the London population.

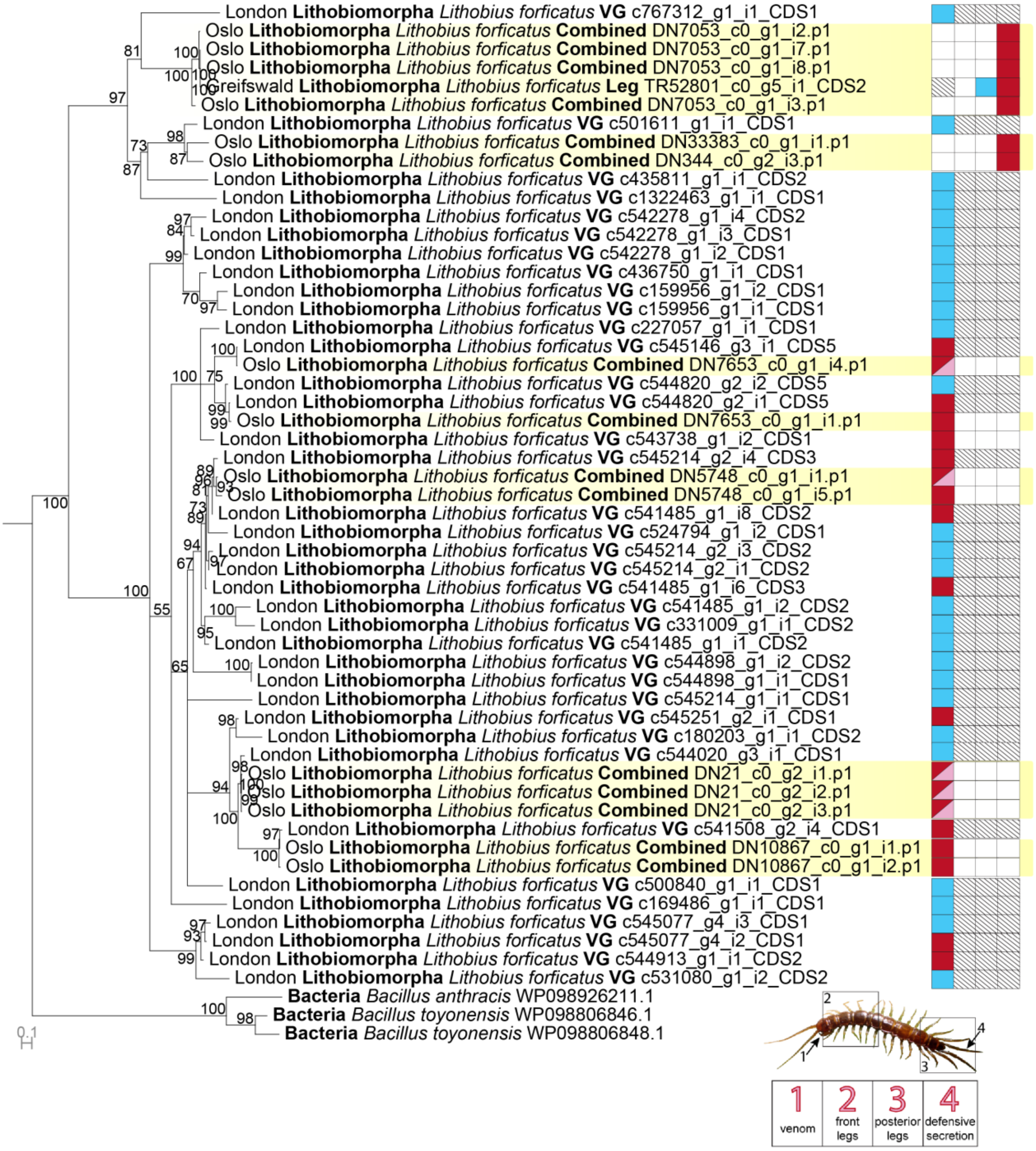

Figure S17. Phylogenetic reconstructions of PCPDPLP sequences found in centipedes. The maximum likelihood reconstructed (under WAG+F+G4) consensus tree is shown as midpoint rooted, while nodes with bootstrap support values < 50 are collapsed. *L. forficatus* sequences from this study are highlighted in yellow. Boxes behind sequences indicate the sequences or absence of each in the (1) venom, (2) front legs (legs 1–4), (3) posterior legs (legs 12–15), or (4) ultimate leg telopodal secretion. Red indicates presence in proteome, blue indicates presence in transcriptome (London and Greifswald populations), pink indicates overexpression (Oslo population), while shaded indicates absence of data.

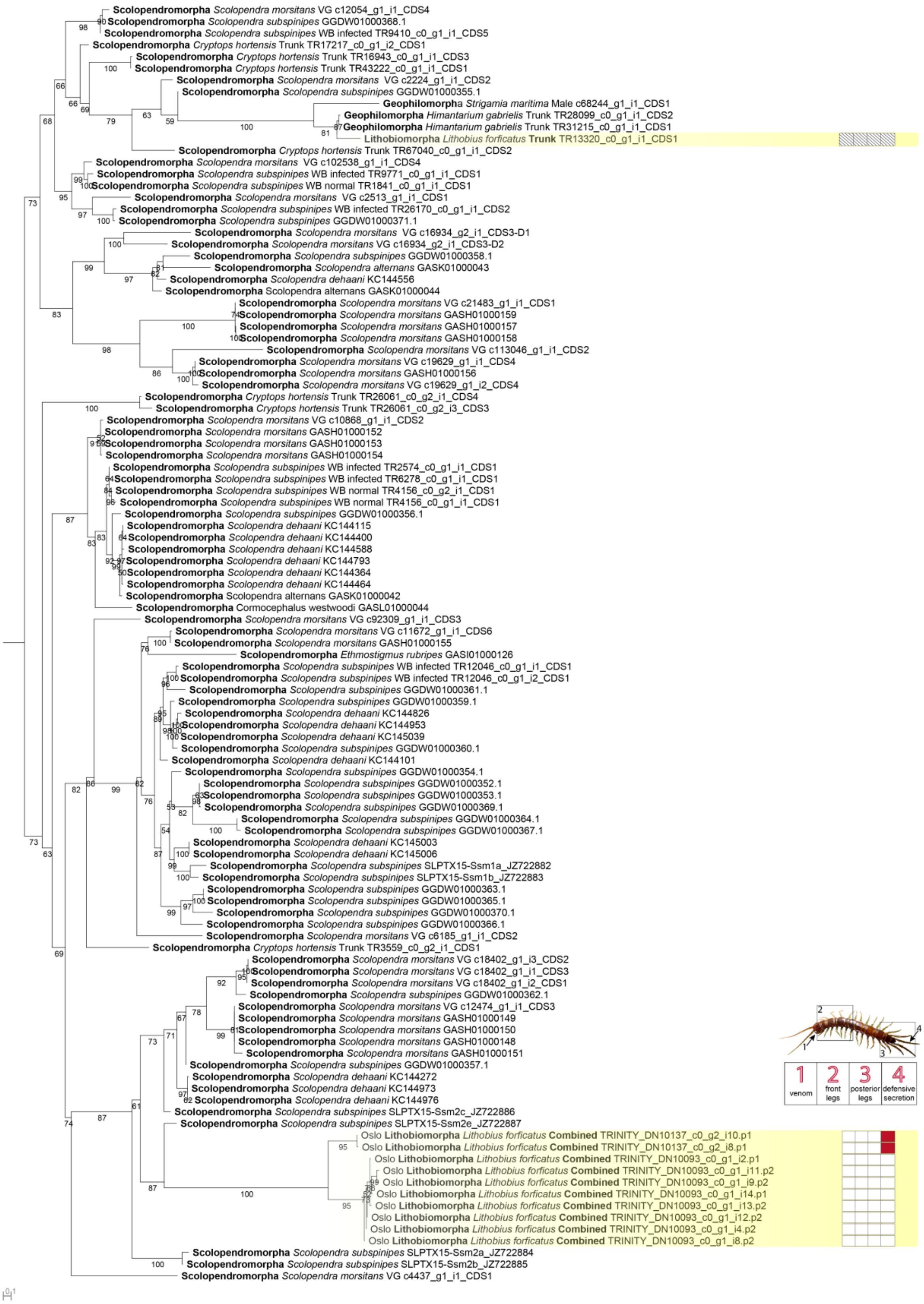

Figure S18. Phylogenetic reconstructions of SLPTX15 sequences found in centipedes. The maximum likelihood reconstructed (under VT+I+I+R3) consensus tree is shown as midpoint rooted, while nodes with bootstrap support values < 50 are collapsed. *L. forficatus* sequences from this study are highlighted in yellow. Boxes behind sequences indicate the sequences or absence of each in the (1)

venom, (2) front legs (legs 1–4), (3) posterior legs (legs 12–15), or (4) ultimate leg telopodal secretion. Red indicates presence in proteome, blue indicates presence in transcriptome (London and Greifswald populations), pink indicates overexpression (Oslo population), while shaded indicates absence of data.
